# The Role of Cation-Vacancies for the Electronic and Optical Properties of Aluminosilicate Imogolite Nanotubes: A Non-local, Linear-Response TDDFT Study

**DOI:** 10.3389/fchem.2019.00210

**Published:** 2019-04-10

**Authors:** Emiliano Poli, Joshua D. Elliott, Sergey K. Chulkov, Matthew B. Watkins, Gilberto Teobaldi

**Affiliations:** ^1^The Abdus Salam Center for Theoretical Physics, Condensed Matter and Statistical Physics Department, Trieste, Italy; ^2^Dipartimento di Fisica e Astronomia “Galileo Galilei”, Università degli Studi di Padova, Padova, Italy; ^3^CNR-IOM DEMOCRITOS, Consiglio Nazionale delle Ricerche-Istituto Officina dei Materiali, Trieste, Italy; ^4^School of Chemical Engineering and Analytical Science, The University of Manchester, Manchester, United Kingdom; ^5^School of Mathematics and Physics, University of Lincoln, Brayford Pool, Lincoln, United Kingdom; ^6^Daresbury Laboratory, Scientific Computing Department, Science and Technology Facilities Council, Warrington, United Kingdom; ^7^Beijing Computational Science Research Centre, Beijing, China; ^8^Stephenson Institute for Renewable Energy and Department of Chemistry, University of Liverpool, Liverpool, United Kingdom

**Keywords:** inorganic nanotubes, imogolite nanotubes, defects, DFT, TD-DFT, optical properties, photo-catalysis

## Abstract

We report a combined non-local (PBE-TC-LRC) Density Functional Theory (DFT) and linear-response time-dependent DFT (LR-TDDFT) study of the structural, electronic, and optical properties of the cation-vacancy based defects in aluminosilicate (AlSi) imogolite nanotubes (Imo-NTs) that have been recently proposed on the basis of Nuclear Magnetic Resonance (NMR) experiments. Following numerical determination of the smallest AlSi Imo-NT model capable of accommodating the defect-induced relaxation with negligible finite-size errors, we analyse the defect-induced structural deformations in the NTs and ensuing changes in the NTs' electronic structure. The NMR-derived defects are found to introduce both shallow and deep occupied states in the pristine NTs' band gap (BG). These BG states are found to be highly localized at the defect site. No empty defect-state is modeled for any of the considered systems. LR-TDDFT simulation of the defects reveal increased low-energy optical absorbance for all but one defects, with the appearance of optically active excitations at energies lower than for the defect-free NT. These results enable interpretation of the low-energy tail in the experimental UV-vis spectra for AlSi NTs as being due to the defects. Finally, the PBE-TC-LRC-approximated exciton binding energy for the defects' optical transitions is found to be substantially lower (up to 0.8 eV) than for the pristine defect-free NT's excitations (1.1 eV).

## Introduction

By altering the local structure and potential experienced by electrons in insulating or semiconducting materials, point-defects such as atomic-vacancies or interstitial atoms can dramatically affect the physical and chemical properties of the defective host material. Control and tuning of the properties brought about by the presence of defects rest on accurate understanding of their properties at the atomic-scale, which has long motivated strong efforts toward the development and application of accurate, yet computationally viable, approaches for the simulation of defects in solids (Shluger et al., 2003; Ganduglia-Pirovano et al., 2007; Lany and Zunger, 2008; Neugebauer et al., 2014; Walsh and Zunger, 2017; Yin et al., 2018).

The ever-increasing demand for sustainable energy keeps driving research in the development of alternative sustainable technologies for energy production and its efficient use. Photo-catalytic materials (photo-catalysts, PCs) are central to these efforts since they can exploit solar light to access alternative, highly selective, reaction routes for fuels and chemicals production (Kamat, 2007; Sastre et al., 2011, 2012a,b,c; Dietl et al., 2012; de Richter et al., 2013; Maeda, 2013; Baltrusaitis et al., 2014; Fresno et al., 2014; Ismail and Bahnemann, 2014), or environmental remediation (Fujishima et al., 2008; Pelaez et al., 2012; Habisreutinger et al., 2013; Navalón et al., 2013). In this context, the search for affordable high performance, highly selective PCs is an ongoing quest, with growing interest in and appreciation of the potential of aluminosilicate and silicate substrates for selective photo-catalysis (Sastre et al., 2012a,b; Alarcos et al., 2017; Murcia-López et al., 2017). As for any other material, also the ground- and excited-state properties of aluminosilicate and silicate-PCs can be significantly affected by the unavoidable presence of defects (Barrer, 1982; Novatski et al., 2008; Tandia et al., 2011).

The growing research in the photo-catalytic potential of 3D nano- and meso-porous silicates (Sastre et al., 2012a,b; Alarcos et al., 2017; Murcia-López et al., 2017), and parallel substantial progress in the synthesis and characterization of 1D-structured aluminosilicate (AlSi) nanotubes (NTs) based on the imogolite (Imo) structure (Wada et al., 1979; Barron et al., 1982; Theng et al., 1982; Mukherjee et al., 2005, 2007; Levard et al., 2008, 2010; Kang et al., 2010, 2011, 2014; Bottero et al., 2011; Yucelen et al., 2011, 2012b; Zanzottera et al., 2012a,b; Bonelli et al., 2013; Amara et al., 2015; Monet et al., 2018), have prompted recent exploratory computational studies of the potential of Imo-NTs for photo-catalytic applications (Teobaldi et al., 2009; Zhao et al., 2009; Poli et al., 2015, 2016; Elliott et al., 2016).

**Figure 1:**
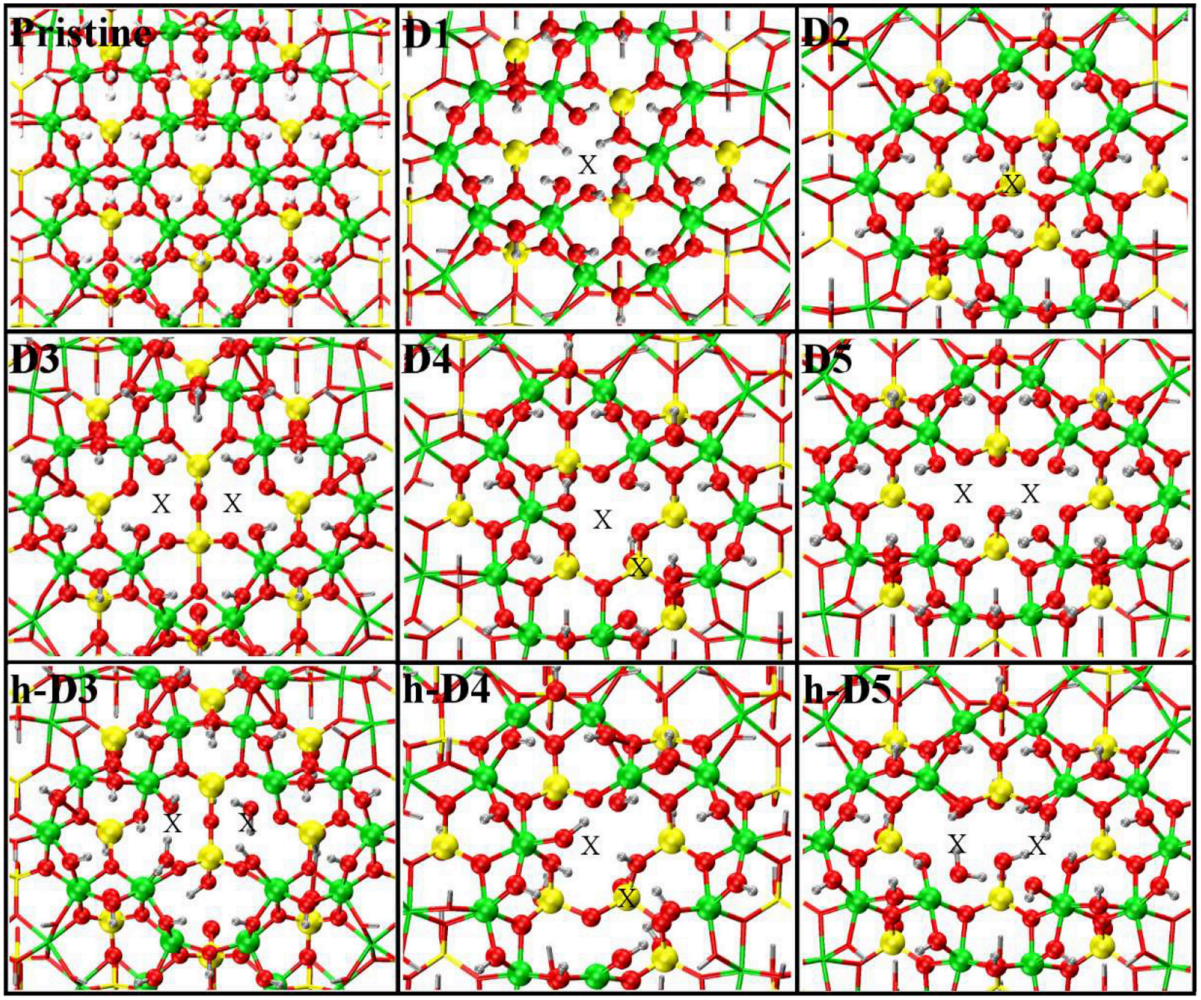
Close up of the atomic structures for the pristine NT, the defects proposed in Yucelen et al. (2012a) (D1–D5), and the protonated defects h-D3, h-D4, and h-D5. Al, green; Si, yellow; O, red; H, silver. An X marker has been used to indicate the position of the missing (Al or Si) atom in the pristine lattice and of the additional Si atoms.

AlSi Imo-NTs are structurally analogous to the naturally occurring hydrous-aluminosilicate imogolite (Cradwick et al., 1972). Their walls consist of a single layer of octahedrally coordinated aluminum hydroxide with pendant tetrahedral silanol (Si–OH) groups facing the tube cavity (Figure 1). The stoichiometric formula of the unit cell is (Al_2_SiO_7_H_4_)_N_, with N being the number of radially non-equivalent aluminum atoms along the NT circumference (an even number for symmetry reasons). In recent years, control over the structure and functionalization of Imo-NTs has improved greatly. Solution-based synthetic routes to produce single-walled AlSi NTs of controllable radius and length have been defined (Wada et al., 1979; Barron et al., 1982; Theng et al., 1982; Mukherjee et al., 2005, 2007; Levard et al., 2008, 2010; Kang et al., 2010; Yucelen et al., 2011, 2012b) and important post-synthetic, selective functionalization of the outer or inner surface of AlSi NTs (Levard et al., 2010; Bottero et al., 2011; Kang et al., 2011, 2014; Zanzottera et al., 2012a,b; Bonelli et al., 2013; Amara et al., 2015) reported. Further progress has been made in the direct synthesis of methylated (AlSi-Me) (Bottero et al., 2011; Bonelli et al., 2013) or aminated (AlSi-Am) (Kang et al., 2014) AlSi NT derivatives, with methyl (–CH_3_) or amine (–NH_2_) moieties in the NT cavity as well as in the creation of hybrid methylated Al(Si/Ge)-Me NTs with tuneable Si/Ge ratio (Amara et al., 2015). Selective amination of the outer surface of AlSi-Me NTs has also been reported (Zanzottera et al., 2012a,b). This synthetic flexibility in turn entails the possibility of widely tuning the NT's properties. Work along this line is ongoing and has led to observation of very encouraging performances of Imo-NTs for chemical separation (Levard et al., 2010; Bottero et al., 2011; Kang et al., 2011, 2014; Zanzottera et al., 2012a,b; Bonelli et al., 2013; Amara et al., 2015), as support for PCs (Katsumata et al., 2013) and as component in hybrid nanocomposites (Marzan and Philipse, 1994). In addition, recent progress in the synthesis of Fe-doped AlSi (and aluminogermanate) NTs (Ma et al., 2012; Arancibia-Miranda et al., 2014; Avellan et al., 2014; Castro et al., 2016; Shafia et al., 2016) has succeeded in introducing absorption of visible (and UV) light, opening new avenues for exploration of the potential of Imo-NTs for both UV and visible light photo-catalytic applications. Recent advances in quantitative structural resolution for (single-walled) Imo-NTs by X-ray scattering (Monet et al., 2018) is expected to further accelerate progress in the development of technological applications based on Imo-NTs. A recent review on Imo-NTs can be found in Paineau (2018).

In spite of the progress in the synthesis and sample-averaged characterization of Imo-NTs (Wada et al., 1979; Barron et al., 1982; Theng et al., 1982; Mukherjee et al., 2005, 2007; Levard et al., 2008, 2010; Kang et al., 2010, 2011, 2014; Bottero et al., 2011; Yucelen et al., 2011, 2012b; Zanzottera et al., 2012a,b; Bonelli et al., 2013; Amara et al., 2015; Monet et al., 2018; Paineau, 2018), the current understanding of point-defects in Imo-NTs is far from complete. To the best of our knowledge, the only experimental investigation of point defects in AlSi Imo-NTs reported to date appears in Yucelen et al. (2012a). By combining different NMR techniques, the authors were able to identify five dominant defect-structures in the AlSi NTs. As explained in Yucelen et al. (2012a), these are based on cation (Al or Si) vacancies, cation (Al → Si) substitution, additional non-stoichiometric Si atoms, and combinations of vacancies with additional atoms (see Figure 1 for the atomistic models of these defects). Analysis of the experimental results led to the estimation that roughly 16% of the Si atoms in the AlSi NTs are involved in a defect structure. Given the size of Imo-NTs and the challenges in accurate modeling of the proposed defects at the experimental concentrations, to date, these defects have never been studied at Density Functional Theory (DFT) level. Although force-field studies of these defects have appeared in the literature (Liou and Kang, 2016), elucidation of their role for the (defective) AlSi NTs' electronic and optical properties is yet to be accomplished. To this end, and to sustain the growing interest in the optical properties of Imo-NTs and their Fe-doped variants (Arancibia-Miranda et al., 2014; Avellan et al., 2014; Shafia et al., 2015, 2016; Castro et al., 2016), here we present a computational study of the role of the NMR-inferred defect structures for the optical and electronic properties of AlSi NTs.

Compromising between AlSi NTs' challenging size, the necessity of simulating models sufficiently large to enable full relaxation of the defects and their coexistence with unperturbed lattice regions in the NT-models as well as the far from solved challenges in accurate simulation of excited states properties for solid-state systems (Gonze et al., 1995; Ghosez et al., 1997; Onida et al., 2002; Bernasconi et al., 2011, 2012; Tomic et al., 2014; Ullrich and Yang, 2014; Casida and Huix-Rotllant, 2016; Strand et al., 2019), here we turn to a recent implementation (Strand et al., 2019) of a non-local [PBE0-TC-LRC (Guidon et al., 2009)], linear-response [LR (Casida, 2009)] time-dependent DFT (TDDFT) approach in the Tamn-Dancoff approximation [TDA (Hirata and Head-Gordon, 1999)], that enables computationally viable LR-TDA-TDDFT simulation for model systems of roughly 1,000 atoms. Although of finite accuracy, especially for the optical properties of defect-free NTs (vide infra), the approach provides an arguably reasonable and timely first step in the elucidation of the optical properties of Imo NTs and defects therein, contributing to research in Imo-NTs and, more generally, to the ongoing work on accurate yet viable simulation of excite-state properties in defective solids, NTs included.

The paper is organized as follows: after presentation of the atomic composition and structure for the NMR-inferred defects and computational methods used (section Methods), we present our results in section Results and Discussion. First, we numerically determine the smallest simulation cell capable of accommodating the defect-induced distortions in the presence of effectively unperturbed lattice regions in the NT model (section Determination of the Supercell Size). Structural analysis of the optimized models is presented in section Optimized Defect Structures, followed by characterization of their electronic properties (section Electronic Structure of the Defective NTs). The calculated optical absorption properties are presented in section Optical properties of defects and discussed with respect to available experimental UV-vis spectra for AlSi NTs. We finally present our conclusions in section Conclusions.

## Methods

### The Atomic Structure of the NMR-Inferred Defects

For our computational study, we started from the atomic structure of the five main defects inferred from NMR experiments on the AlSi Imo-NTs (Yucelen et al., 2012a). They can be seen in Figure 1 and are described and labeled as follows:

- **D1** is a single Al-vacancy. By disrupting the gibbsite Al(OH)_3_ backbone of the NT, D1 introduces additional Al-OH dangling groups in the NTs framework, which would otherwise not be present in the defect-free NTs.
- **D2** presents an additional Si-atom in the inner wall of the NTs, leading to the occurrence of a Si-O-Si bridge in the inner wall of the NTs. It is worth recalling that the pristine NT do not contain Si-O-Si bridges, but only pendant silanol (Si-OH) groups.
- **D3** is a double Al-vacancy in the outer gibbsite layer of the NT. NMR data (Yucelen et al., 2012a) suggests the condensation of two Si-OH groups with formation of an *elongated* Si-O-Si bridge, which we also included in our initial geometry ahead of geometry optimization. In the absence of structural relaxation for the rest of the NT-atoms, the elongated nature of the bridge leads to an unusually large Si-O-Si angle of 169° and partially stretched (1.73 Å) Si-O bond lengths.
- **D4** comprises one Al-vacancy in the outer wall, and one extra Si-atom in the inner wall of the NTs. As for D2 and D3, also D4 contains an additional Si-O-Si bridge not present in the pristine NT structure.
- **D5** consists of two neighboring Al-vacancies. In contrast to D3, no Si-O-Si bridge is present, leaving two Si-OH groups dangling around the double-vacancy site.

Table 1 reports the stoichiometry of the defect models for the adopted x3 periodic repeat of the NT (vide infra, section Determination of the Supercell Size). Given the water-phase synthesis of the AlSi NTs (Mukherjee et al., 2005, 2007; Levard et al., 2008), it is inevitable that counter-cations or protons (in the water solution) will compensate the negative charge of D3, D4, and D5. These considerations prompted us to model three additional structures, one for each negatively charged defect, neutralizing the original negative excess charge by protonation of the defect-site. These structures, labeled **h-D3**, **h-D4** and **h-D5** are also displayed in Figure 1. Inclusion in the simulation of larger counter-cations [possibly Keggins ions as experimentally observed during the NT synthesis (Casey, 2006)] was not considered due to the prohibitive computational cost of this approach.

**Table 1:** Stoichiometric formula and resulting charge for the considered defect models.

**#Defect**	**Model stoichiometry**	**Net charge**
D1	(Al_2_SiO_7_H_4_)_71_SiAlO_7_H_7_	0
D2	(Al_2_SiO_7_H_4_)_71_Si_2_AlO_8_H_5_	0
D3	(Al_2_SiO_7_H_4_)_71_SiO_5_H_2_	−4
D4	(Al_2_SiO_7_H_4_)_71_Si_2_O_7_H_4_	−2
D5	(Al_2_SiO_7_H_4_)_71_SiO_6_H_4_	−4
h-D3	(Al_2_SiO_7_H_4_)_71_SiO_5_H_6_	0
h-D4	(Al_2_SiO_7_H_4_)_71_Si_2_O_7_H_6_	0
h-D5	(Al_2_SiO_7_H_4_)_71_SiO_6_H_8_	0

### Computational Methods

#### Geometry Optimization

Given the extended dimension of the systems considered (~1,000 atoms) all the defect structures were optimized using the Linear Scaling DFT program ONETEP (Skylaris et al., 2005; Haynes et al., 2006; Hine et al., 2009, 2011) with the PBE (Perdew et al., 1996) approximation to the exchange-correlation (XC) functional, and separable (Kleinman-Bylander) norm-conserving pseudopotentials (Gonze et al., 1991) for the atomic core levels. The adopted kinetic energy cutoff was 1,000 eV, and 4 (9) valence Non-Orthogonal Wannier Functions (NGWFs) were used for the O (Al, Si) atoms. 1 NGWF was used for the H atoms. In all cases, no truncation of the density kernel (*K*^αβ^) was enforced. The localization radius for the valence NGWFs was 8 Bohr. All simulations were performed with periodic boundary conditions ensuring at least 15 Å vacuum separation between replicated images along the non-periodic directions. Along the periodic direction of the NT, the simulation cell contained three-fold (x3) replicas of the NT along its axis (optimized 8.665 Å period), yielding models of ~1,000 atoms. This choice is extensively discussed and motivated in section Determination of the Supercell Size. Geometry-relaxations were performed via the quasi-Newton optimization scheme based on the Broyden-Fletcher-Goldfarb-Shanno (BFGS) algorithm (Pfrommer et al., 1997). All the atoms of the defective NT-models were left free to relax. The geometry optimization threshold for the atomic forces was 0.05 eV/Å. Consistent with the absence of any dangling bonds (vide infra), singlet spin-states were tested to be energetically favored over alternative spin-polarized solutions.

#### Hubbard-Corrected, Non-local DFT, and LR-TDDFT Simulations

Following geometry relaxation, the optimized defect models were used for single-point non-local hybrid DFT and LR-TDDFT simulations using the CP2K package (VandeVondele et al., 2005). CP2K uses a mixed Gaussian and plane wave basis set, where atom-centered Gaussian orbitals are used to represent the wavefunction, while the electronic density is represented by an expansion of plane waves.

Through all our calculations, we used Goedecker-Teter-Hutter (GTH) norm-conserving pseudopotentials (Goedecker et al., 1996) to model the electron-ion interactions. We adopted the molecularly optimized (MOLOPT) DZVP-SR basis set (VandeVondele and Hutter, 2007) for all atoms and the auxiliary density matrix method (ADMM) (Guidon et al., 2010) to compute the exchange integrals, using the cpFIT3 basis set for all the elements present in our systems.

Hubbard-corrected (PBE+U) simulations were also performed with the CP2K code applying an isotropic U = 7 eV correction on the 2p subspace of the O-atoms. The *U*-value was taken from earlier benchmarked studies of Al-doped bulk SiO2 (Nolan and Watson, 2006; Mao et al., 2017).

For hybrid DFT and LR-TDDFT simulations, we used the PBE0-TC-LRC XC-functional (Guidon et al., 2009) with 25% Hartree-Fock (HF) exact-exchange mixing and a real-space cut-off of 8 Å, beyond which a long-range correction [based on the spherically averaged PBE exchange hole (Ernzerhof and Perdew, 1998)] is used.

Optical spectra were computed both at the independent-particle level making use of the Fermi Golden Rule (Read and Needs, 1991; Motta et al., 2010) and via the linear-response (LR) time-dependent DFT (TDDFT) formalism (Casida, 2009) recently implemented, in the Tamn-Dancoff approximation [TDA (Casida, 2009)], in CP2K by two authors of the present paper (Strand et al., 2019). In all cases, the first 60 virtual states were converged for each defect structure using a cutoff of 500 Ry. The absorption spectra were reproduced using a Gaussian broadening of 0.3 eV. Consistent with the closed-shell ground-state for the considered systems, and based on the expected extremely low probability of a singlet to triplet transition due to spectroscopic selection rules, only singlet excitation were considered in the LR-TDA-TDDFT calculations. The real space cutoff of 8 Å was numerically tested to best compromise between (i) convergence in excitation energies (50 meV) and absorption intensities (5%) sufficient to discriminate between the main features in the simulated optical spectra, and (ii) practical viability of the simulations on Tier0 High Performance Computing resources (Figures S1, S2 in the Supporting Information).

Depending on the choice of the method used to simulate optical absorption, different quantities were calculated and are accordingly displayed. For the FGR approach, we computed the imaginary part of the dielectric function (ε_2_). Conversely, for the LR-TDA-TDDFT approach, we computed the absorbance (e, L mol^−1^ cm^−1^) from the calculated oscillator strengths for each optical excitation following the standard procedure reported in http://gaussian.com/uvvisplot/.

## Results and Discussion

### Determination of the Supercell Size

When simulating defects by means of periodic DFT simulation approaches as used here, the size of the periodic cell containing the defect is one of the critical aspects to be numerically checked. To reduce the computational cost without introducing (finite-size) errors in the simulations, the simulation cell should be as small as possible yet capable to preserve bulk-like, unperturbed regions around the defect site following structural relaxation.

For this initial benchmark, we focused on D1. The optimized structure of the AlSi NT in Yucelen et al. (2012a) (24 Al-atoms in the circumference, 336 atoms in total) was modified to recover the same D1 composition and structure derived from the NMR study in Yucelen et al. (2012a). The same procedure was repeated for two larger systems made up by two (672 atoms) and three (1,008 atoms) NT-repeat units, respectively.

**Figure 2:**
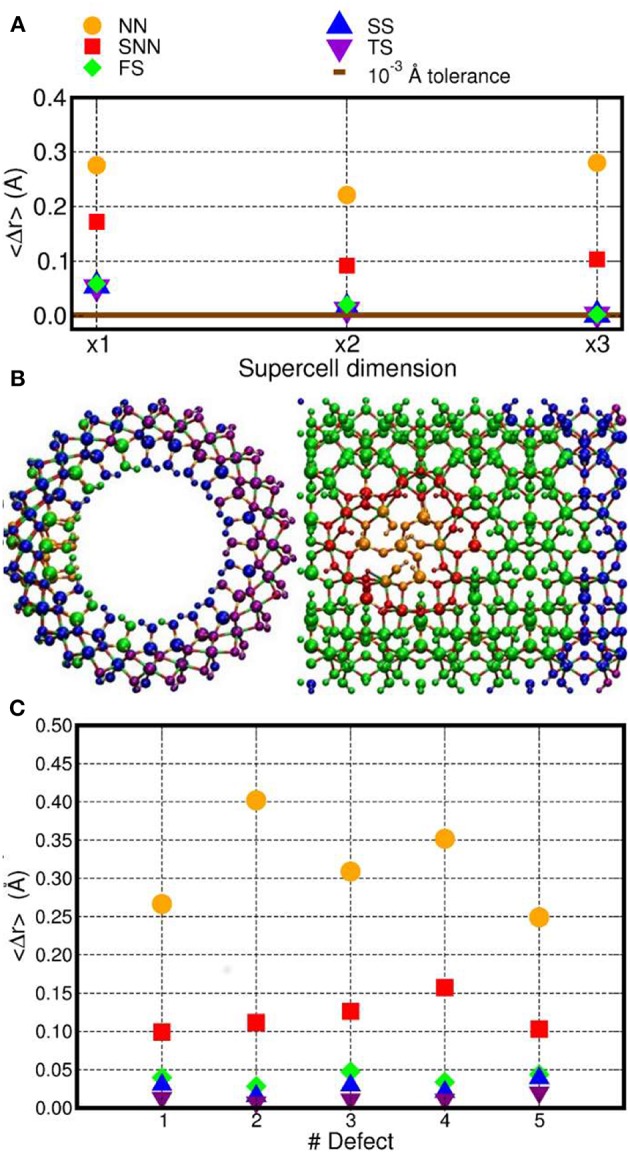
**(A)** Computed average atomic-displacements from initial bulk-like positions (〈Δ*r*〉, Å) for the optimized D1 model as a function of the number of NT-repeat units in the simulation cell (supercell dimension). See text for the definition of the NN, SNN, FS, SS, and TS labeling for the different group of atoms as a function of their distance from the center of the D1 defect. **(B)** Front and side view of the x3 AlSi NT model hosting D1. The atoms have been colored as a function of their 〈Δ*r*〉 value. Same color labeling as in **(A)**. **(C)** Computed 〈Δ*r*〉 for the D1–D5 models (x3 supercell).

The structure of these three differently sized models of D1 was optimized by fully relaxing all the atoms in the simulation cells. We then analyzed the defect-induced atomic-displacements as a function of the distance from the defect site (Figure 2A). For a suitably sized model, the regions furthest from the defect site should maintain the same atomic structure as in the perfect, defect-free, system.

To facilitate the analysis, the NT-atoms were divided in different groups based on their distance from the defect-center (Figure 2). The atoms directly bonded to a missing atom in the defect structures are referred to as nearest neighbors **(NN)**. The atoms separated by one bond from the NN atoms are marked second nearest neighbors **(SNN)**. The atoms within a 10 Å distance from the defect-sites (but further from the defect center than the SNN atoms) have been inserted in a group called first shell **(FS)**. Accordingly, the FS group does not include NN and SNN atoms. The same approach has been used for atoms within a 15 Å (second shell, **SS**) and 20 Å (third shell, **TS**) distance from the center of the defect-site. The computed displacements from the initial bulk-like positions were then group-averaged, obtaining the 〈Δ*r*〉 values shown in Figure 2.

The simulation-cell containing two AlSi NT repeat units shows small but non-negligible displacements for the SS and TS atoms (〈Δ*r*〉~10^−2^ Å. Conversely, the supercell containing three AlSi NT repeat-units presents negligible 〈Δ*r*〉 ≤ 10^−3^ Å values for all the (FS, SS and TS) atoms further than the SNN group. The extremely small displacements for the TS atoms demonstrates that the simulation cell with three NT repeat units suffer from very limited (i.e., negligible) finite-size effects and enable modeling of regions of the defective NT with a pristine unperturbed structure.

The same analysis was repeated also for all the others defects in Table 1 using the largest x3 simulation cell. The results are shown in Figure 2B. Exception made for D3, the average relaxation-displacements for the atoms further than the SNN (i.e., >15 Å away from the defect site) are in the order of 10^−2^ Å or less for all defects D2 to D5. The larger 〈Δ*r*〉 values for D3 can be ascribed to its more extended structure (Figure 1), leading to a longer-range perturbation of the host lattice. The computed 〈Δ*r*〉 < 10^−2^ Å value for the TS atoms (further than 20 Å from the defect center) of D3, nevertheless suggest rather contained finite-size effects also for this defect, when modeled in a x3 supercell of the AlSi NT.

Overall, the results in Figure 2A demonstrates that, for atoms belonging to the TS groups (hence furthest from the defect-site within the periodic simulation cell), the defect-induced perturbation are very effectively screened, leading to negligible displacements from bulk-like positions. On the basis of these results, a x3 simulation cell with three NT repeat units was used for the electronic structure characterization. This choice permits to simulate defects concentrations involving ~10% of the Si-atoms in the cells (for D1), which slightly underestimates the experimentally determined value of 16% (Yucelen et al., 2012a). Accordingly, and pending limitations of the adopted PBE XC-functional and neglect of solvent in the simulations, the considered models should provide a reasonable representation of the defects in AlSi NTs.

### Optimized Defect Structures

The atomic structure for defects D1 to D5 in Yucelen et al. (2012a) were obtained from a combination of several NMR techniques (“^1^H–^29^Si and ^1^H–^27^Al FSLG-HETCOR, ^1^H CRAMPS, and ^1^H–^29^Si CP/MAS”), *without* further refinement via energy-based geometry optimization. In the following, we accordingly analyse in detail the role of (DFT) energy-based geometry optimization (in vacuo) for the atomic structure of the defects D1 to D5 both qualitatively and quantitatively.

**Figure 3:**
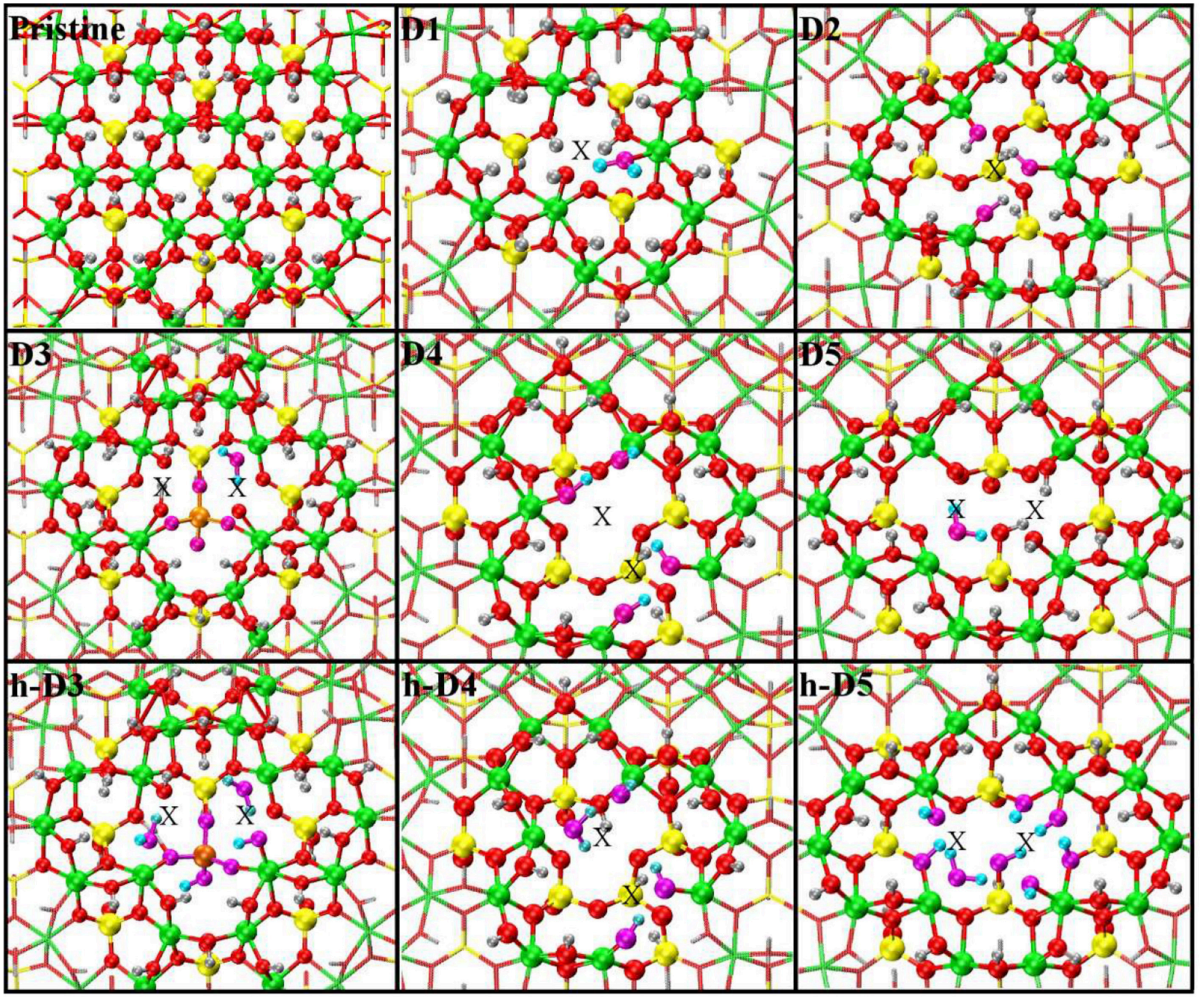
Close up of the optimized atomic structures for the pristine NT, the defects proposed in Yucelen et al. (2012a) (D1–D5), and the protonated defects h-D3, h-D4, and h-D5. Al, green; Si, yellow; O, red; H, silver. To highlight changes from the initial structures in Figure 1, an alternative atom-coloring has been used for the dangling -OH groups closest to the defect sites, condensed H_2_O molecules, perturbed hydrogen-bonding patterns, and the Si-O-Si bridge in D3 (O, magenta; H, cyan; Si, tangerine). An X marker has been used to indicate the position of the missing (Al or Si) atom in the pristine lattice and of the additional Si atoms.

The optimized structures for the D1-5 and h-D3-5 defects are shown in Figure 3. The D1 optimized structure presents a water (H_2_O) molecule coordinated to an Al-atom adjacent to the defect site. The formation (*condensation*) of one Al-coordinated H_2_O molecule is due to the transfer of one H-atom from a dangling –OH group to an adjacent one. As for D1, also optimization of D5 leads to condensation of one H_2_O molecule that remains coordinated to one of the Al-atoms closest to the center of the defect-site.

Optimization of the D2 structure leads to local re-organization of the local hydrogen-bond network both inside and outside the NT-cavity with no H_2_O condensation. A qualitatively similar relaxation (rearrangement of inner and outer hydrogen bonding network with no H_2_O condensation) is found also for D4.

Also for D3, the optimized geometry presents noticeable differences with respect to the NMR proposed structure in Yucelen et al. (2012a). Upon DFT-relaxation, the stretched Si-O-Si bridge breaks and the O-atom relaxes toward the closest Si-atom leaving a negatively charged deprotonated O-atom. The computed barrier-less breaking of the originally elongated Si-O-Si bridge is accompanied by extensive reorganization of the AlO_6_ octahedron closest to the defect site. This relaxation in turn leads to condensation of a H_2_O molecule, which remains coordinated to one Al-atom with additional hydrogen bonding coordination (1.53 Å distance) to one deprotonated O-atom from a neighboring Al-octahedron. This geometry was found to be energetically favored by at least 0.4 eV over the alternative geometries studied (Figure S3 in the Supporting Information).

The final structure for the protonated defects (h-D3, h-D4, h-D5) are relatively similar to their charged correspondent. However, the presence of additional hydrogen-atoms around the defect sites leads to condensation of an additional H_2_O molecule. In all cases, the formed H_2_O molecules remain coordinated the Al-atoms nearest to the center of the defects.

Given the strong sensitivity to proton environments in the experiments in Yucelen et al. (2012a), it is possible the deviation between the NMR proposed structure (Figure 1) and DFT-optimized one for D1 and D3 (Figure 3) may be due to the lack of solvent (H_2_O) and/or counterions in the simulations. The prohibitive computational (memory) cost of simulating the present models in the presence of explicit solvation, and the unavailability of DFT-benchmarked force-fields for defects in AlSi NTs, prevent us from further investigating this aspect, that is however worth of specialist method development work.

**Figure 4:**
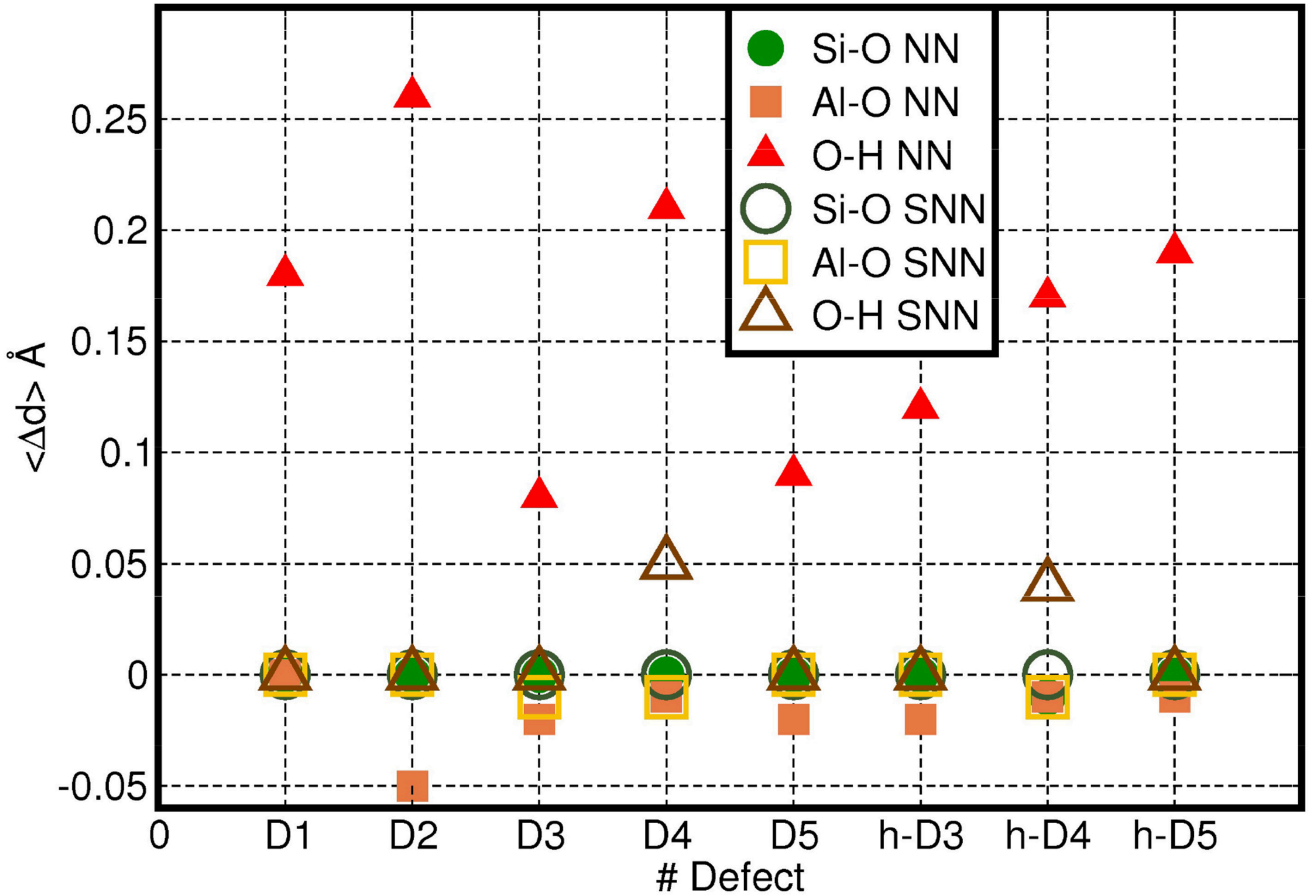
Computed average change in Al-O, Si-O, and O-H bond-lengths (〈Δ*d*〉, Å) for the NN (filled symbols) and SNN (empty symbols) atoms as a function of the defect-model studied. The adopted reference for each bond type is reported in Table S1 in the Supporting Information.

To provide a more quantitative analysis of the optimized defect structure, we next consider their effects on the three types (Si-O, Al-O, O-H) of bonds in AlSi NTs. Given the computed small average-displacement (〈Δ*r*〉) for atoms further than the FS group (Figure 2), we limit the present analysis to the atoms belonging to the NN and SNN groups. Figure 4 reports the computed average change in Al-O, Si-O, and O-H bond-lengths (〈Δ*d*〉) for the atoms in the NN and SNN groups i.e., those closest to the defect-center. The raw data for this analysis can be found in the Supporting Information (Table S1).

In general, the presence of cation vacancies in the AlSi NTs framework causes larger changes in the external Al-O octahedral layer than in the inner Si tetrahedrons. The change in length of the Al-O bonds for the NN, SNN atoms varies from case to case. Whereas, the computed deviations for the Al-O bond-lengths in the NN and SNN groups lie in the 3–10% range (~0.02 − 0.15 Å), those calculated for the stronger Si-O bonds are consistently smaller than 0.01 Å.

Owing to the creation of alternative local hydrogen-bonding patterns and/or condensation of H_2_O (D2, D4, h-D4. h-D5 in Figure 3) the occurrence of these defects in the NTs induces substantially larger relaxations (up to 〈Δ*d*〉 >0.25 Å values in Figure 4).

It is useful to note that the modeled barrier-less H_2_O condensation is found to take place only in defect structures for which the O-O distance between adjacent Al-OH dangling group or between an Al-OH dangling group and a pristine Al-O(H)-Al bridge is <2.5 Å. In D2, D4, and h-D4 the presence of an extra Si atom increases the distances between the closest dangling Al-OH groups. Provided this condition is not fulfilled, no barrier-less H_2_O condensation takes places during the geometry optimization. However, and interestingly, the absence of H_2_O condensation during the relaxation of D2, D4, and h-D4 is shown in Figure 4 to lead to an overall larger structural relaxation around the defect. Altogether, these results suggest that H_2_O condensation in non-stoichiometric defective AlSi NTs is an effective mechanism to reduce the spatial-extent of perturbation to the NT-lattice, thence strain.

### Electronic Structure of the Defective NTs

As noted above, to the best of our knowledge, the electronic properties of the NMR-inferred defects in Yucelen et al. (2012a) have not been previously considered in the available literature on imogolite, which motivates our interests in the subject and this section. Based on (i) the negligible defect-induced structural distortion computed for the NT-regions farthest from the defect-sites (Figure 2), suggesting coexistence of both defect-sites and nearly unperturbed NT-regions in our models, and (ii) the encouraging accuracy of PBE0-related XC-functionals in calculating Kohn-Sham BGs (Guidon et al., 2009, 2010), we expect our results to provide a reasonable approximation of the real NTs (in vacuo).

**Figure 5:**
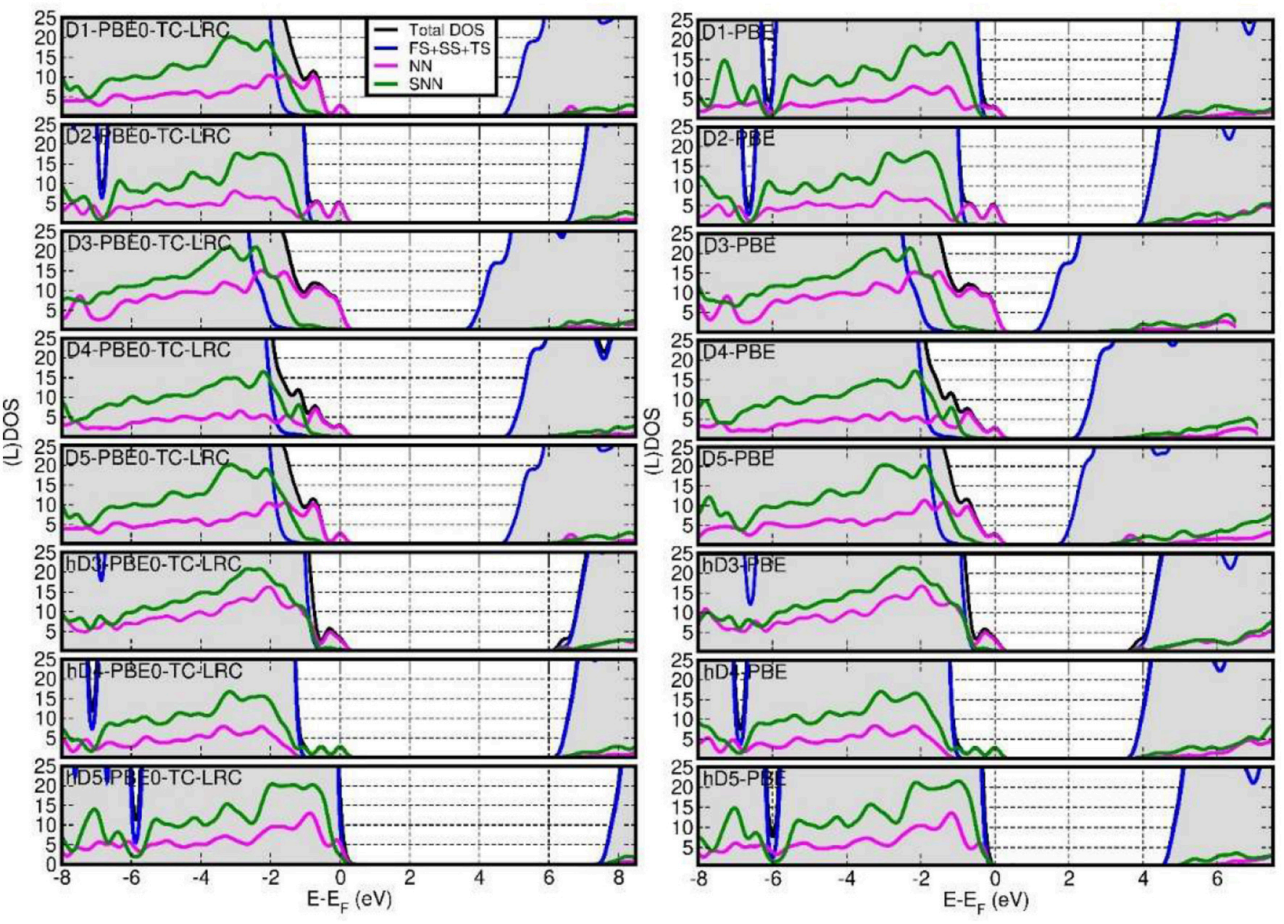
Fermi-energy (E_F_) aligned PBE0-TC-LRC **(left)** and PBE **(right)** DOS for all the defects simulated. NN-, SNN- and (FS+SS+TS) resolved local-DOS (LDOS) are also displayed. The energies have been referenced to the highest occupied Kohn-Sham states and the (L)DOS have broadened via 0.1 eV Gaussian smearing. All the computed systems are insulating with no fractional occupancy: occurrence of LDOS traces beyond E_F_ is due to the applied smearing.

Figure 5 reports the computed total Density of States (DOS) and defect-site resolved local DOS (LDOS) at both PBE and PBE0-TC-LRC levels. Depending on the defect and its protonation, shallow (h-D5) or deep (D1, D2, D3, D4, D5,h-D3, h-D4) occupied states are introduced in the NT's BG. The ensuing reduction of the energy gap between the highest-energy occupied state and lowest-energy empty state is found to be highly sensitive to the defect composition and structure, with values ranging from 0.25 eV (h-D5) to up to 4.12 eV (D3).

Comparison between the results for the D3-5 defects and their hydrogenated counterparts (h-D3-5) indicates that protonation is effective in turning the deep defect-states of (D3-5) into shallower ones (h-D3-5), with changes as large as 1.5 eV going from D5 to h-D5. For D4, protonation of the defect site leads to shift of the defect states closer to the Valence Band edge and on the SNN atoms. These results hint at the possibility of controlling the energy of the defect states, thence the NT's electronic properties, by altering the pH of the solution the NTs are dissolved in. Although, the absence of implicit or explicit solvent (and control of pH conditions) in our simulations prevents us from drawing firm conclusions on this subject, this result may motivate further experimental research in the pH-dependence of Imo-NTs' optical spectra [not considered in Shafia et al. (2016) and Shafia et al. (2015)].

**Figure 6:**
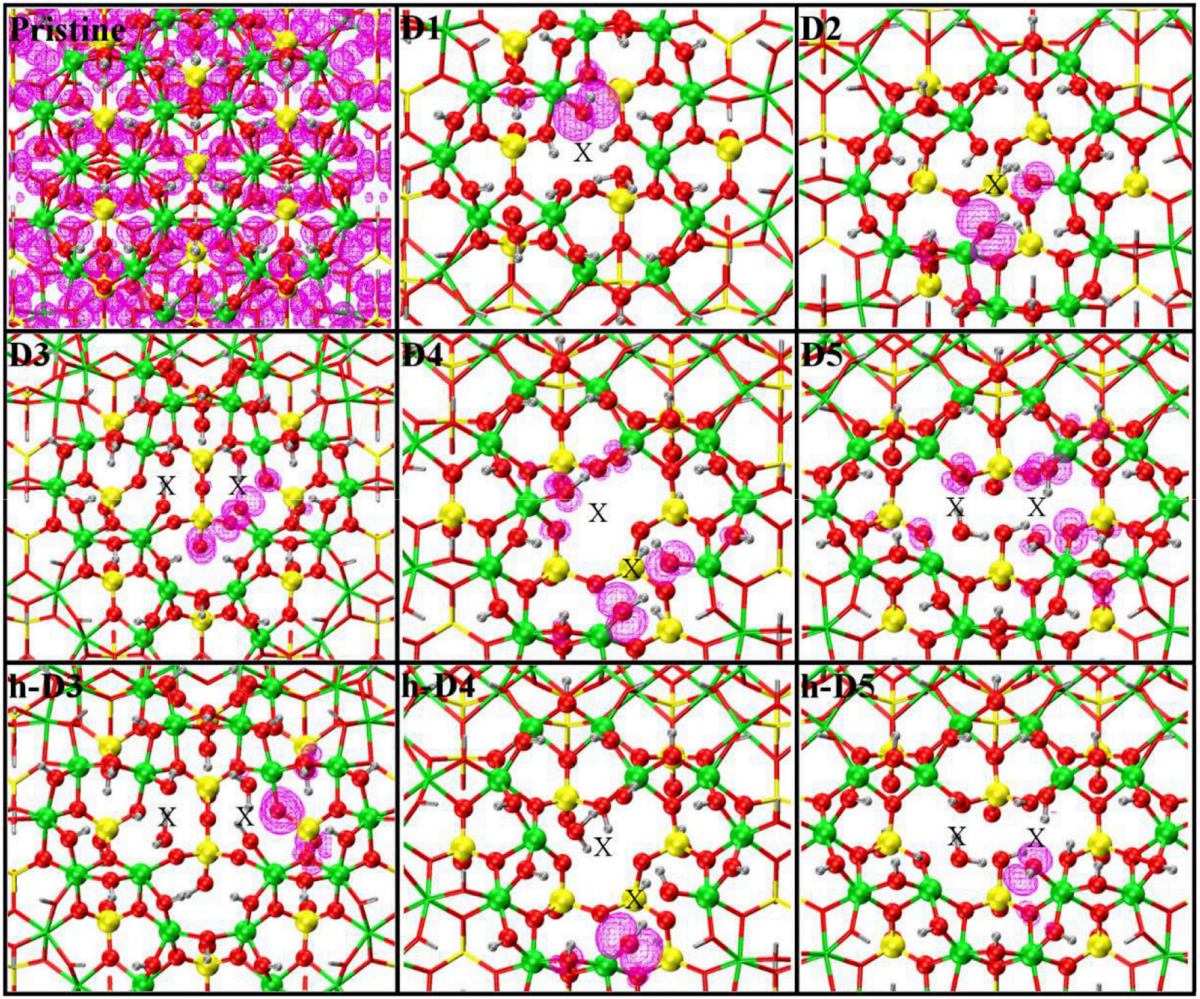
Close up of the optimized defect geometries together with the density-plot of their highest-energy occupied Kohn-Sham states (magenta). Same atomic color labeling as in Figure 1. An X marker has been used to indicate the position of the missing (Al or Si) atom in the pristine lattice and of the additional Si atoms.

In all cases, the computed LDOS traces indicate dominant contributions to the occupied defect-states from the NN and SNN atoms closest to the center of the defects. Thus, as shown in Figure 6, the occupied defect-states turn out to be highly localized at (or around) the defect site. Conversely, the computed (L)DOS indicate that, for all cases, the CB-edge remains delocalized on the unperturbed NT, being dominated by contributions from the atoms furthest from the defect-sites.

**Figure 7:**
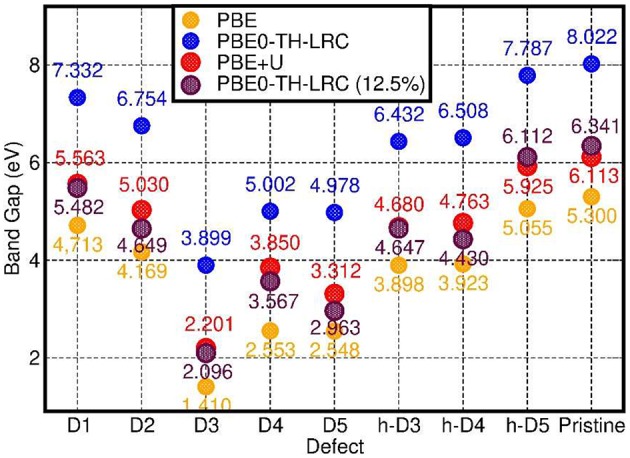
Comparison between the calculated BGs for the considered systems at PBE, PBE0-TH-LRC, PBE+U, and PBE0-TH-LRC (12.5% HF mixing) level.

In spite of quantitatively different BG values (Figure 7), comparison between the PBE, PBE0-TC-LRC (25 and 12.5% HF exact-exchange mixing) and PBE+U calculated LDOS (Figure 5 and Figures S4–S7 in the Supporting Information) reiterates a rather weak dependence of the contributions to the VB-edge and defect-states on the given approximation to the XC-functional. A partial exception to this trend is represented by application of 7 eV Hubbard corrections on the O-atoms (2p subspace) of D2 and h-D5, which results in a slightly enhanced localization of the defect-states. In spite of these small differences, all the computational approaches used agree in predicting the defect states localized at the defect-site.

To further support this statement, we re-optimized the structure for two selected systems, namely the single Al-vacancy (D1) and the mixed Al-vacancy/additional Si system (D4) at both PBE0-TC-LRC and PBE+U = 7 eV level. New Figure S8 in the Supporting information provides a visual comparison between the PBE, PBE+U and PBE0-TC-LRC optimized geometries for D1 and D4. Figure S9 in the Supporting Information provides a quantification of the deviations of the PBE+U and PBE0-TC-LRC optimized geometries from the PBE-optimized one.

With average deviations smaller than 0.15 Å even for the Nearest Neighbors (NN) atoms (that are directly bonded to a missing/additional atom), the PBE0-TC-LRC geometries are found to be in good agreement with PBE results. As shown in Figure S8 the deviations are mostly due to small changes in bond-orientations as well as hydrogen bonding patterns. Conversely, use of a rather aggressive U = 7 eV correction for the 2p subspace of the O-atoms is found to induce somewhat larger differences between PBE and PBE+U optimized geometries for D1 and D4 (up to ~0.25 Å average displacement for the NN atoms of D4). The substantially larger (~0.35 Å) average displacement for the NN atoms of D1 at PBE+U level is due to the absence of water condensation for the PBE+U optimized geometry, in contrast with PBE and PBE0-TC-LRC results.

As the U = 7 eV O(2p) correction was parameterized on experimental data for hole band-gap states in Al-doped SiO_2_ (Nolan and Watson, 2006; Mao et al., 2017), a system not containing Al-OH or Al-OH_2_ fragments, we are inclined to consider the agreement between PBE and PBE0-TC-LRC results (leading to water condensation) as a robust benchmark of the initially proposed PBE results. Regardless of these specialist and perhaps secondary differences, the good agreement between the differently calculated (L)DOS (Figure 5 and Figures S4, S5, S10) and real-space localization plots (Figure 6 and Figures S6, S7, S11) for D1 and D4 reiterates the conclusion that, pending minor quantitative difference, the physical picture provided by PBE0-TC-LRC refinement of PBE-optimized geometry offer a good compromise between absence of major artifacts and computational costs, at least for the systems considered here.

Altogether, the marked localization of the occupied defect-states, and the computed delocalization of the CB-edge (Figures 5, 6) hint to the possibility of defect-mediated separation of photo-generated e-h pairs via energetically advantageous relaxation of holes to different defect-sites in the NTs. Thus, and pending the actual electron (hole) transfer kinetics which may hinder such separation process, the present results suggest that cation-vacancies in Imo-NTs could be highly reactive photo-oxidation centers. In addition, the presence of occupied defects states in the BG for defective NTs strengthens earlier suggestions (Levard et al., 2010; Yucelen et al., 2012b) regarding the potential of Imo-NTs as hole-scavengers for molecular photo-catalysts grafted on the NT's wall.

Finally, for the specialist reader we quantify the deviations between PBE and PB0-TC-LRC results for the BGs of the considered systems. As shown in Figure 7, not unexpectedly, the PBE-calculated BGs are substantially smaller than the PB0-TC-LRC ones with deviations in the 2.43-2.73 eV range depending on the defect. However, and in spite of the expected underestimation of BGs, the calculated relative energies and amplitudes of the PBE LDOS traces, especially for the occupied states, are in very good agreement with the PB0-TC-LRC ones (Figure 5). This result points to a reasonable performance of the PBE functional for the simulation of the relative energy- and real-space localization of occupied cation-vacancy states in Imo-NTs. Not unexpectedly, the results for 12.5% HF mixing are found to lie in between the PBE and PBE0-TH-LRC (25% HF mixing) values. Interestingly, BG values for 12.5% HF mixing appear to be very similar to the PBE+U = 7 eV results with relatively limited (300 meV) system-dependent deviations between the two datasets.

As shown in Figure 7, for the pristine NTs, the calculated BG is 5.3 eV and 8.0 eV at PBE and PB0-TC-LRC level, respectively. The calculated PBE value of 5.3 eV is in quantitative agreement with earlier results at a similar level of theory (Zhao et al., 2009), and underestimated with respect to the expectedly more accurate PB0-TC-LRC value. Experimentally, aluminosilicate NTs [with 16% of Si atoms involved in defects (Yucelen et al., 2012a)] are observed to start to absorb light at roughly 4.1 eV (300 nm) with a low-energy absorbance shoulder at roughly 5 eV (250 nm) preceding a marked increase in the absorbance at 5.5–6.2 eV (225–200 nm) (Shafia et al., 2015). Tauc-plot processing of the experimental spectra leads to suggestion of an “optical gap” of 4.9 eV for the pristine (direct gap Zhao et al., 2009, Elliott et al., 2016) NTs (Shafia et al., 2015). The marked overestimation (3.1 eV) of the PB0-TC-LRC BG with respect to the low-energy features in the experimental absorption spectra point to possibly crucial roles of both existing defects (Yucelen et al., 2012a) and excitonic relaxations (Ullrich and Yang, 2014) for the optical properties of aluminosilicate NTs. In the following Section we investigate both these points.

### Optical Properties of Defects

In spite of the growing interest in imogolite NTs, characterization of their optical properties has not been as systematic as the study of their synthesis and potential for chemical separation. To the best of our knowledge, the only UV-vis optical characterization of purified (pristine and Fe-doped) imogolite NTs' samples to date have been published in Shafia et al. (2015) and Shafia et al. (2016). Also to the best of our knowledge, simulation of the optical properties of imogolite nanotubes beyond the independent-particle Fermi Golden Rule (FGR) approximation [with the PBE functional (Elliott et al., 2016; Poli et al., 2016)] is yet to be explored in the literature. To contribute to this knowledge gap, we next investigate the optical properties of the defective NTs-models at different level of theories, analyzing in detail the role of the XC-functional (PBE vs. PBE0-TC-LRC) and optical excitation approach (FGR vs. LR-TDA-TDDFT) for the simulated results.

**Figure 8:**
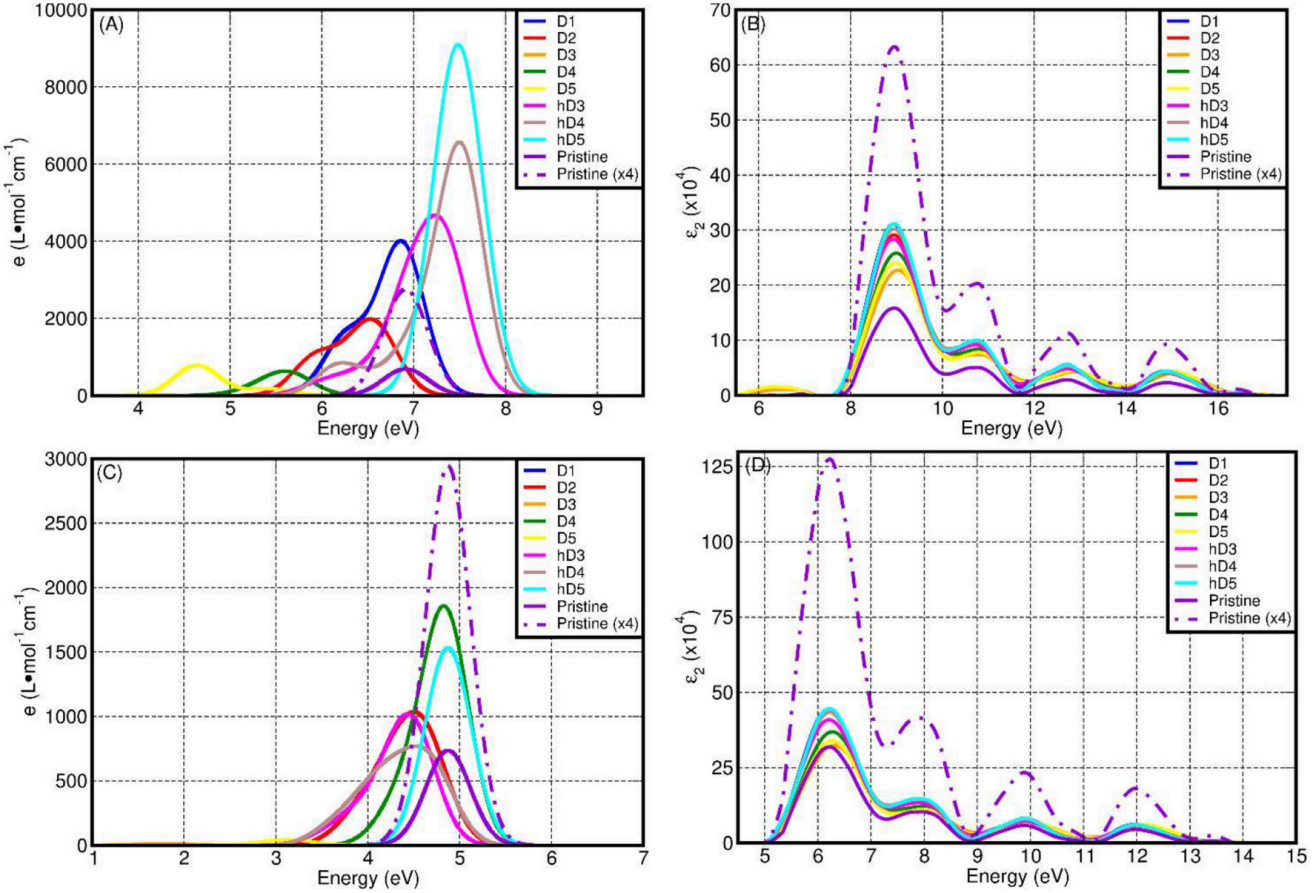
Computed LR-TDA-TDDFT optical spectra at PBE0-TC-LRC **(A)** and PBE **(C)** level of theory for all the systems considered, together with the corresponding FGR spectra, also at PBE0-TH-LRC **(B)** and PBE **(D)** level. Owing to the substantially lower computed absorbance, the D3 traces in **(A,C)** cannot be resolved for the adopted scale of the y-axis.

Figure 8 reports a comparison between the LR-TDA-TDDFT and FGR spectra computed at both PBE0-TC-LRC and PBE level for all the considered defects. We start our analysis from the LR-TDA-TDDFT spectra at PBE0-TC-LRC level that have been computed with that the least aggressive approximations among all the different combinations presented. All the neutral system (pristine NT, D1, D2, h-D3, h-D4, and h-D5) are computed to present a main absorption peak between 6.5 and 7.5 eV. Conversely, for the negatively charged defects (D3, D4, D5), we compute absorbance peaks at lower energy, with D5 presenting a ~4.5 eV absorption peak. Interestingly, depending on the chemical composition (and charge) of the defect, the computed absorbance peak can be both red-shifted (D1, D2, D3, D4, D5) and blue-shifted (h-D3, h-D4, h-D5) with respect to the defect-free NT's absorbance.

Comparison between the calculated spectra suggests a larger low-energy optical absorbance with respect to the pristine NTs for all the defective structures but D3, whose low-energy absorbance is computed to be strongly suppressed with respect to the defect-free NT (Tables S5, S11 in the Supporting Information). Thus, the simulations suggest defective NTs sections to absorb more light than defect-free sections. As shown in Figure 8A, the calculated absorbance for D1, h-D3, h-D4, h-D5 is 10–20 times larger than for the defect-free NTs. Instead, for D2, D5, and D4 the absorbance is twice or comparable to the defect-free NT. Although an immediate connection between the local chemical bonding in the defects and the value of the absorbance cannot be easily drawn, it is interesting to note that protonation of D3, D4, and D5 defects (likely for the defective NTs in water solution at slightly acidic pH as the defect is negatively charged) results in increased optical absorbance. We believe these results motivate further experimental research in the pH-dependence of Imo-NTs's optical properties, an aspect not considered in Shafia et al. (2016) and Shafia et al. (2015).

Before discussing the results of the simulations against published experiments, we recall that as per the NMR study of Yucelen et al. (2012a), up to 16% of Si atoms are involved in a cation vacancy defect and that defects D1 to D5 should be simultaneously present in the NTs. That is, defect-free NTs are not expected to exist, at least for the currently available synthetic routes. We also recall that, experimentally, AlSi NTs start to absorb light at roughly 4.1 eV (300 nm) with a low-energy absorbance shoulder at roughly 5 eV (250 nm) preceding a marked increase in the absorbance for energies larger than 5.5–6.2 eV (225–200 nm) (Shafia et al., 2015).

Based on the computed PBE0-TC-LRC, LR-TDA-TDDFT spectra in Figure 8A, it is tempting to tentatively interpret the shape of the experimental spectra in the 300–200 nm (4.1–6.2 eV) region as being due to convolution of the progressively larger absorbance peaks of D5, D4, D2, and D1, with the defect-free NTs contributing -but not dominating- the absorbance around 7 eV. As a direct consequence of the present results, the Tauc-plot derived value of 4.9 eV should likely be re-assigned to a strong defect absorbance (D5), rather than the intrinsic optical-gap of pristine nanotubes (calculated to be a ~7.0 eV with an approximated –likely underestimated– exciton binding energy of ~1.1 eV, see below). Given the computed sensitivity to protonation for the absorbance of defects D3 to D5, further experimental research in the dependence of the NTs' UV-vis spectra on the pH conditions would be highly desirable to validate (or confute) the PBE0-TC-LRC, LR-TDA-TDDFT predictions. Recording of spectra below 200 nm would be also highly desirable and strongly contribute to the ongoing work for development of TDDFT strategies to excitons in solids (Gonze et al., 1995; Ghosez et al., 1997; Bernasconi et al., 2011; Tomic et al., 2014; Ullrich and Yang, 2014).

**Figure 9:**
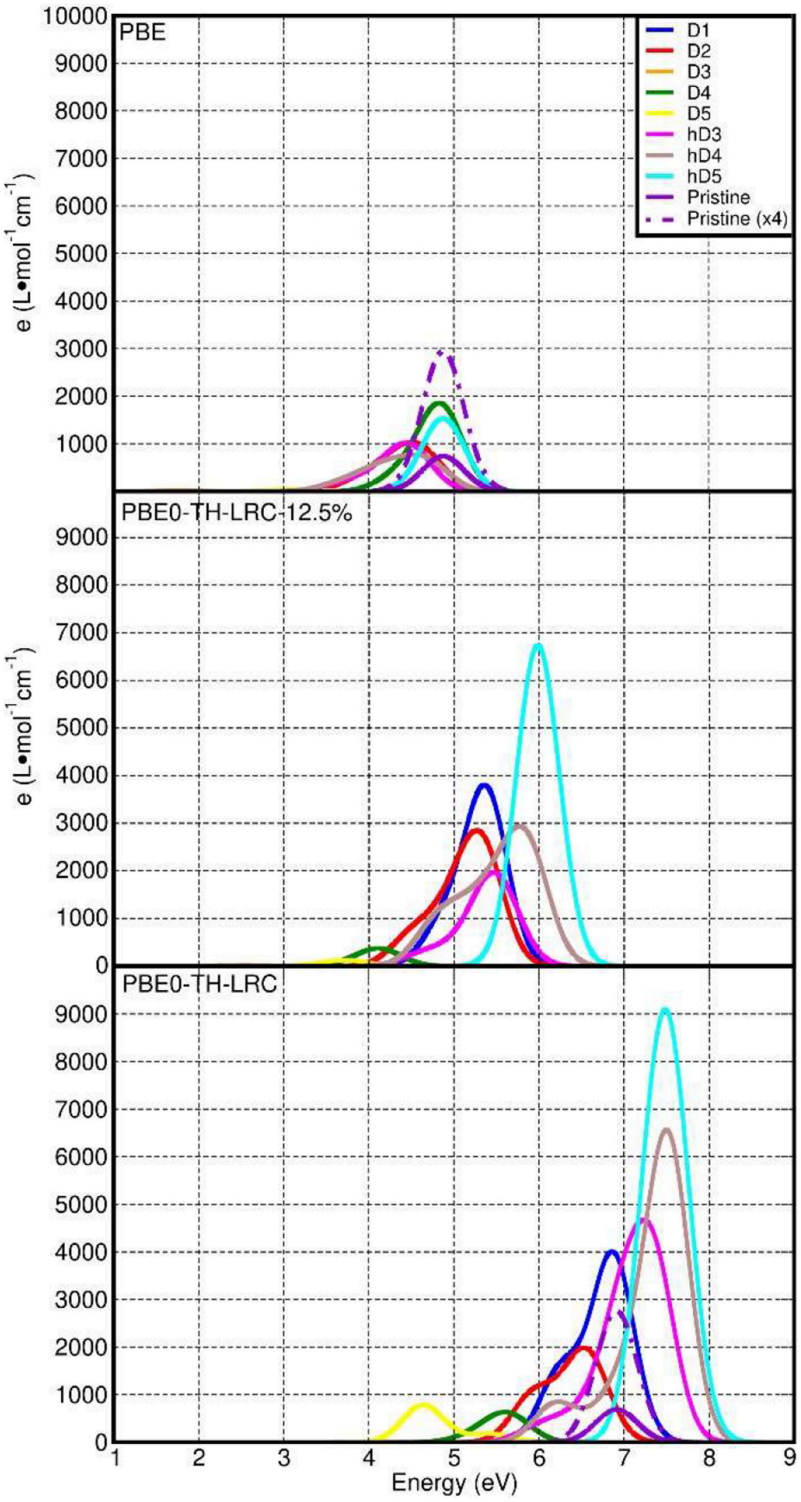
Comparison between the computed LR-TDA-TDDFT optical spectra at PBE **(top)**, PBE0-TH-LRC (12.5% HF mixing, **middle**) and PBE0-TH-LRC (25% HF mixing, **bottom**) level of theory for all the systems considered. The use of the same scale for the x- and y-axis to favor comparison leads to some PBE and PBE0-TH-LRC (12.5% HF mixing) traces being not visible in the plots.

We have also quantified the role of HF mixing (25% as per original PBE0-TC-LRC and 12.5%) for the calculated LR-TD-TDDFT optical properties. To this end, Figure 9 reports a comparison between the PBE, PBE0-TC-LRC (12.5% HF mixing), and PBE0-TC-LRC (25% HF mixing). In general, reduction of the HF mixing from the original 25–12.5% leads to a decrease of both the energy and intensity of the optical absorption peaks. The relative energy and intensity ranking for the defect considered remains qualitatively unaltered by reduction of the HF mixing. The only exceptions to this trend are the h-D3 and h-D4 systems that, for 12.5% mixing, are not computed to have the 2nd and 3rd largest absorption peak (as for 25% HF mixing). Decrease of HF mixing from 25 to 12.5% is found also to lead to disappearance of a well-defined low-energy shoulder for D1 and D2, indicative of a different energy distribution for the optically active electronic excitations in the system. In spite of the quantitative dependence of the calculated spectra on the HF mixing in the hybrid-DFT approach, the results of this benchmark strengthen our earlier assignment of the experimental low-energy absorption onset [4.1-6.2 eV, (Shafia et al., 2015)] being due to the NMR-inferred defects in the NTs.

We next consider the difference between the calculated PBE0-TC-LRC Kohn-Sham BGs and the lowest energy LR-TDA-TDDFT excitation as a first approximation to the exciton binding energy in the defective NTs. As explained in Ullrich and Yang (2014) (Figure 2 and related discussion), the approximation stems from (i) the PBE0-TC-LRC BG being used instead of the true *quasi-particle gap*, and (ii) the approximated PBE0-TC-LRC description of the many-body e-h interactions leading to stabilization of the exciton (as measured by the exciton binding energy). Therefore, although encouraged by previous successful applications of unscreened non-local XC-functionals (e.g., B3LYP) with non-local long-range terms in the xc-kernel (f_xc_) (Bernasconi et al., 2011, 2012; Tomic et al., 2014; Ullrich and Yang, 2014), a note of caution is in place, especially in the absence of many-body benchmark results on defective Imo-NTs' true quasi-particle gap, a quantity not available in the literature and well beyond our computational resources. Moreover, due to the use of real-space cut-off for the exchange integrals in our simulations at PBE0-TC-LRC (Guidon et al., 2009; Strand et al., 2019), the use of a long-range (semi-local) correction may prevent quantitative description of diffuse excitations leading to highly delocalized electron-hole pairs (Gonze et al., 1995; Ghosez et al., 1997; Ullrich and Yang, 2014). In spite of this limitation, the localized nature of the defect occupied states (dominating the low energy optical excitations, vide infra) and the inclusion of exact-exchange to within a 8 Å, should jointly contribute to contain the deficiencies intrinsic to the adopted approach. As noted in the methods section (see also Figures S1, S2), the adopted 8 Å cutoff was numerically checked to yield excitation energies (~7 eV defect-free NT absorbance-peak included) converged to within 0.05 eV for D1, which, at least to our minds, vouches for a favorable compromise between accuracy and computational viability of the present approach for the system studied.

**Figure 10:**
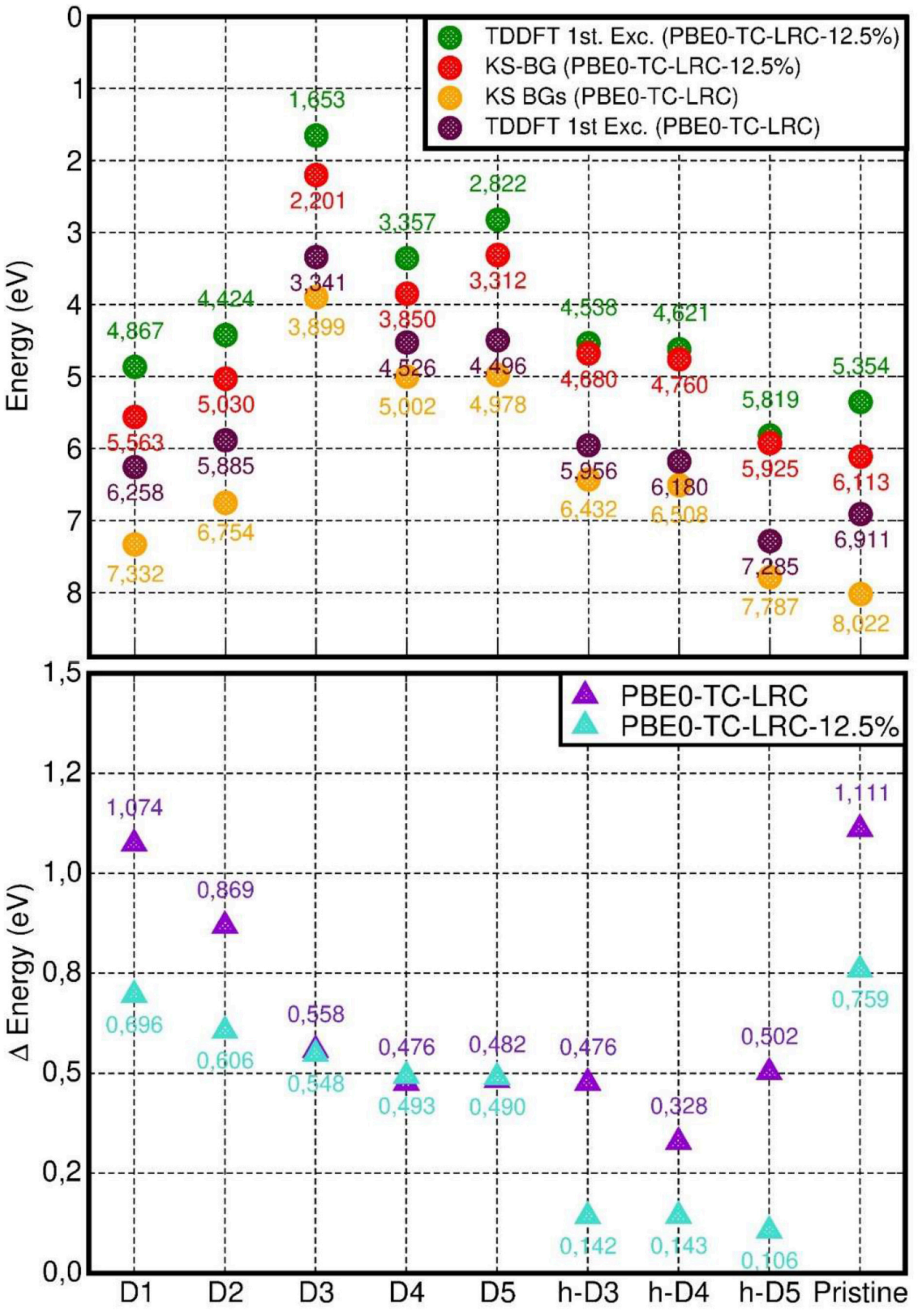
**Top:** computed PBE0-TC-LRC Kohn-Sham BG and LR-TDA-TDDFT lowest energy excitation energy (“1st Exc”). **Bottom:** difference between the calculated Kohn-Sham BG and LR-TDA-TDDFT 1st excitation energy.

As shown in Figure 10, the computed energies differences (approximated exciton binding energies) are consistently larger than 0.3 eV and can be as large as ~1.1 eV for the pristine NT and D1. By comparing the results in Figure 10 with the computed measures of defect-induced structural relaxation (Figure 2), it transpires that, at least for the system studied, the exciton binding energy decreases as a function of the defect-induced structural reorganization (D1: ~1.1 eV, D2: ~0.9 eV, D3-5, and h-D3-5: < 0.6 eV). Thus, disruption of the cylindrical symmetry for the NT, appears to reduce the excitonic binding energy, at least for the modeled systems in vacuo.

Reduction of the HF mixing from 25 (standard PBE0-TH-LRC) to 12.5% leads to a parallel substantial (up to nearly 400 meV for the pristine NT) decrease in the approximated exciton binding energy for all the cases apart from the negatively charged defects (D3, D4, and D5). For the latter cases, the computed differences in approximated exciton binding energies are smaller than 20 meV. This result in turn suggests a reduced role for non-local interactions (as captured by the HF contribution to the XC-functional) for the low-energy excitations of these systems. As also evident from Figure 10, in spite of quantitative differences, use of both 25 and 12.5 % HF mixings consistently leads to smaller (approximated) exciton binding energy for the systems characterized by larger structural relaxation, strengthening the earlier conclusions based on original PBE0-TH-LRC (25% HF mixing) results.

In an attempt to further investigate the nature of the absorbance peaks and low-energy shoulders in Figure 8, we next analyse, for each considered case, the five excitations with the largest oscillator strength. Tables S3–S11 and Figures S12–S20 in the Supporting Information reveal the presence of at least one secondary excitation with non-negligible oscillator strength for D1, D2, and h-D4. These secondary excitations are, respectively 0.62 eV (D1), 0.87 eV (D2), and 1.34 eV (h-D4) lower in energy with respect to the main peak. In spite of the complex composition of calculated absorbance spectrum, the largest oscillator strength excitations in Tables S3–S10 and Figures S12–S19 consistently involve transitions from defect-localized orbitals to delocalized CB orbitals (in general far from the defective part of the NTs). Combined with the substantial reduction (>0.5 eV) in the approximated exciton binding energy for all the NMR-inferred defects but D1 and D2 (Figure 9), it is unavoidable to note that altogether these results point to the possibility of defect-mediated exciton-separation in AlSi NTs, with creation of highly-localized oxidative states at the defect-sites. Given the ongoing experimental work in development of (UV-light) sustainable photo-catalytic strategies for selective production (oxidation) of high-value chemicals (Sastre et al., 2012a,b; Alarcos et al., 2017; Murcia-López et al., 2017), and the absence of experimental results on the excitonic binding energies in Imo-NTs, these results prompt for interest, further research (and validation opportunities) by the experimental community. The current unavailability of excited state gradients in the used implementation of LR-TDA-TDDFT (Strand et al., 2019), prevents us from further exploring this point by simulation.

Finally, for the specialist reader we briefly discuss the dependence of the computed spectra on the simulation protocol. We start by considering the role of the xc-functional for the computed LR-TDA-TDDFT spectra. Due to both the BG underestimation (Figure 7) and the lack of non-local terms in the xc-kernel (f_xc_) (Gonze et al., 1995; Ghosez et al., 1997; Bernasconi et al., 2011, 2012; Tomic et al., 2014; Ullrich and Yang, 2014), the PBE LR-TDA-TDDFT spectra (Figure 8C) deviates from the PBE0-TC-LRC ones both in terms of computed absorbance values (reduced for PBE) and relative energy of the absorbance peaks. Apart from D5, the absorption peak for all the defects and the pristine NT is now computed between 4.2 and 5 eV. The largest (smallest) absorbance is computed for the D1 and D4 (D3 and D5). That is, at odds with the PBE0-TC-LRC results, protonation is not modeled to increase optical absorbance at PBE-level, in contrast with PBE0-TC-LRC results. Table S2 in the Supporting Information reports a comparison between the approximated (LR-TDA-TDDFT) exciton binding energies at PBE0-TC-LRC (see also Figure 10) and PBE level. Apart from the pristine nanotubes, D1 and D2 the approximated (LR-TDA-TDDFT) exciton binding energies are found not to majorly change depending on the use of the semi-local PBE or non-local PBE0-TC-LRC (the computed changes are smaller than ~0.1 eV). This results suggest that depending on the structural reorganization of the defects (smaller for D1 and D2, larger for the other defects in Figure 2), long-range effects become progressively more screened and less important for the electronic and optical properties of Imo-NTs. The agreement between 25 and 12.5% HF mixing results for D3-D5 in Figure 10 strengthens this conclusion.

Regardless of the use of PBE0-TC-LRC or PBE, simulation of the spectra at the independent-particle level via the FGR consistently lead to an absorbance maximum at ~9 (PBE0-TC-LRC) and 6.2 eV (PBE) with qualitatively similar computed spectra. It thus emerges that, regardless of the XC-functional used, application of the FGR prevents the eventual use of the computed absorbance spectra to distinguish between defects and pristine NTs. Accordingly, and not unexpectedly, it transpires that use of the LR-TDA-TDDFT approximation at PBE0-TC-LRC level appears to offer the most favorable accuracy-viability compromise for the study of the optical properties of point-defects in Imo-NTs, at least out of the simulation protocols explored. Given the availability of experimental spectra for Fe-doped aluminosilicate NTs (Shafia et al., 2015, 2016) future work will investigate the performance of the present PBE0-TC-LRC, LR-TDA-TDDFT approach on these systems to sustain research both in the Imo-NTs area and simulation of optical properties in (1D) solids.

## Conclusions

Simulation of the five NMR-inferred defect structures (D1 to D5) for AlSi NTs (Yucelen et al., 2012a) and their protonated counterparts (h-D3 to h-D5) indicates that:

- The presence of defects induces local deformations in the NT structure that can be suitably simulated in models based on a x3 repeat unit of the NTs. The defect-induced deformations affect primarily the outer Al-O(H) octahedrons and the external O-H hydrogen-bonding network.
- All the considered defects leads to the appearance of occupied shallow or deep defect-states highly-localized at the defect-site. The energy-position of these states is computed to be strongly sensitive to protonation of the defect site.
- Owing to the absence of dangling bonds in the considered systems, the occurrence of defect-induced shallow or deep sates is found to be rather insensitive to the use of the PBE, PBE+U = 7 eV, PBE-TC-LRC (25% HF mixing) or BE-TC-LRC (12.5 HF mixing).
- With the exception of D3, the low-energy optical absorbance of the defects is generally substantially (x2-x20) larger than for defect-free NTs. Protonation of the defect sites leads to increase of the optical absorbance.
- Based on the calculated PBE0-TC-LRC, LR-TDA-TDDFT spectra, the experimental low energy (4.1–6.2 eV) absorbance tail for AlSi NTs can be interpreted as due to the convolution of the optical absorbance of the NMR-inferred defects.
- The differences between the calculated PBE0-TC-LRC Kohn-Sham BGs and the lowest energy LR-TDA-TDDFT excitation, a first approximate measure of the exciton binding energies, are found to be systematically reduced from 1.1 (defect-free NT) to <0.5 eV at the defect sites.

Based on the current lack of experimental results on the electronic and optical properties of defects in imogolite NTs, we believe the present results will be a positive complement to research in the field.

## Author Contributions

EP executed the simulations and analyzed the results in collaboration with JE, MW, and GT. SC and MW implemented and tested the used TDDFT formalism in CP2K. EP and GT wrote the initial draft of the manuscript. All the authors contributed to the final version of the manuscript.

### Conflict of Interest Statement

The authors declare that the research was conducted in the absence of any commercial or financial relationships that could be construed as a potential conflict of interest.
