# Supplementary material for: The Role of Cation-Vacancies for the Electronic and Optical Properties of Aluminosilicate Imogolite Nanotubes: A Non-local, Linear-Response TDDFT Study
Source: Front Chem. 2019 Apr 10;7:210. doi: 10.3389/fchem.2019.00210 (PMC6469436; doi:10.3389/fchem.2019.00210)
Supplement: Supplementary file 1 [file Data_Sheet_1.pdf]

Supplementary Material

The role of cation-vacancies for the electronic and optical properties of aluminosilicate imogolite nanotubes: a non-local, linear-response TDDFT study

E. Poli[1,*], J. D. Elliot[2,3,4], S. K. Chulkov[5], M. B. Watkins[5,*], G. Teobaldi[6,7,8,*]

[1]The Abdus Salam Center for Theoretical Physics, Condensed Matter and Statistical Physics Department, I-34151 Trieste, Italy

[2]Dipartimento di Fisica e Astronomia “Galileo Galilei”, Università degli Studi di Padova, I-35131 Padova, Italy

[3]CNR-IOM DEMOCRITOS, Consiglio Nazionale delle Ricerche--Istituto Officina dei Materiali, c/o SISSA, I-34136, Trieste, Italy

[4]School of Chemical Engineering and Analytical Science, The University of Manchester, Manchester M13 9PL, United Kingdom

[5]School of Mathematics and Physics, University of Lincoln, Brayford Pool, Lincoln LN6 7TS, United Kingdom

[6]Scientific Computing Department, Science and Technology Facilities Council, Daresbury Laboratory, Warrington WA4 4AD, United Kingdom

[7]Beijing Computational Science Research Centre, 100193 Beijing, China

[8]Stephenson Institute for Renewable Energy and Department of Chemistry, University of Liverpool, Liverpool L69 3BX, United Kingdom

*** Correspondence:**
Emiliano Poli
epoli@ictp.it

Matthew B. Watkins
MWatkins@lincoln.ac.uk

Gilberto Teobaldi
gilberto.teobaldi@stfc.ac.uk

Keywords: inorganic nanotubes, imogolite nanotubes, defects, DFT, TD-DFT, optical properties, photo-catalysis

1 Supplementary Figures and Tables

1.1 Supplementary Figure

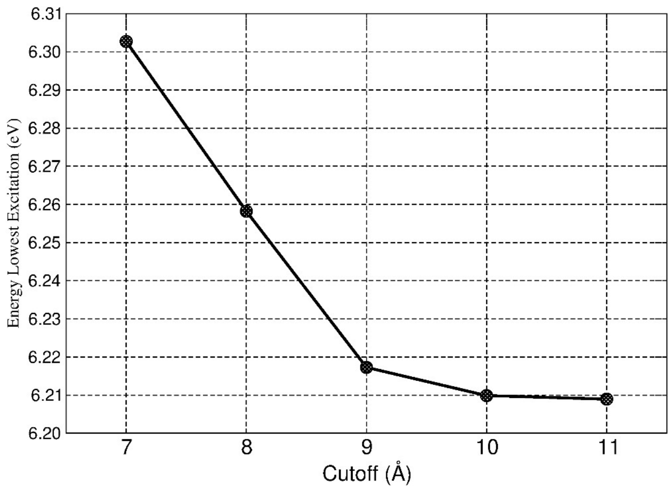

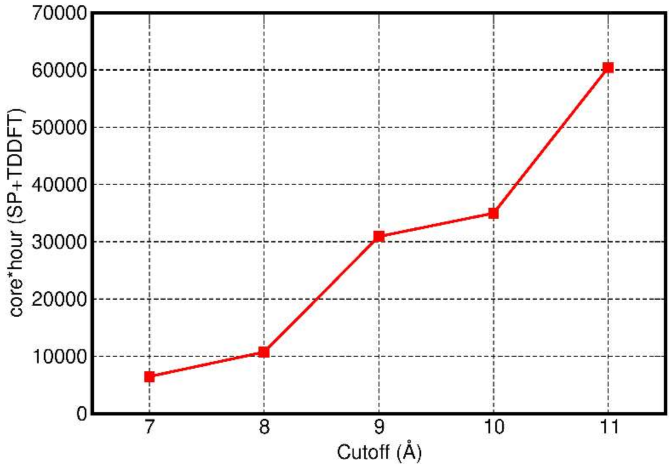

Figure S1. (Top) Calculated LR-TD-TDDFT PBE0-TC-LRC lowest-energy excitation (eV) for **D1** as a function of the PBE0-TC-LRC truncation cutoff (Å). (Bottom) Change in overall computation time (core-hours) for the corresponding single-point (SP) and TDDFT calculation as a function of the PBE0-TC-LRC truncation cutoff (Å). All the simulations were carried out on 15 nodes (720 cores) of the Marconi Tier0 HPC system.

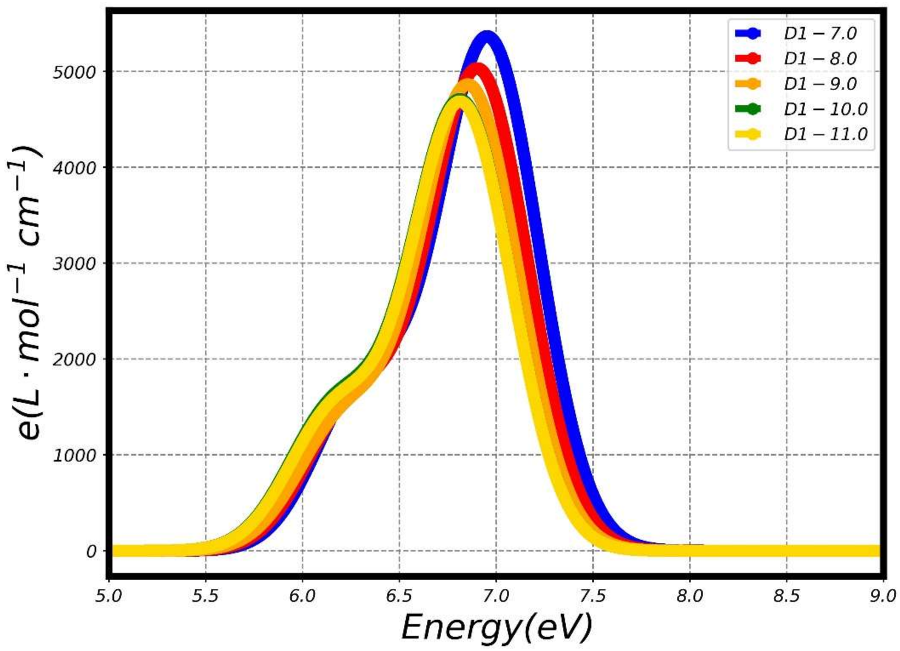

Figure S2. Calculated LR-TD-TDDFT absorption spectra for **D1** as a function of the PBE0-TC-LRC truncation cutoff (Å).

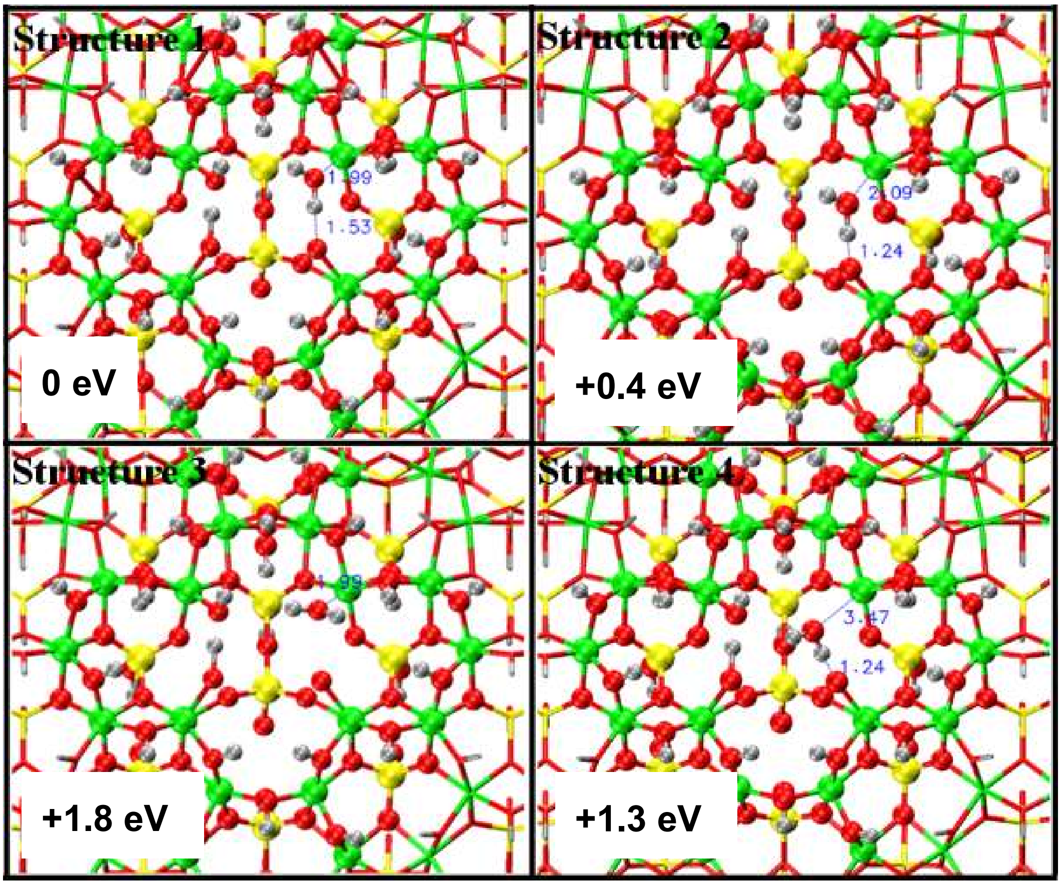

Figure S3. Close up of the four considered competing structures for **D3** with relative PBE energies. The energy favoured Structure 1 is the same as in Fig. 3. Al: green, Si: yellow, O: red, H: silver. Hydrogen bonding distances have been marked by dashed blue lines, with corresponding lengths (Å) reported.

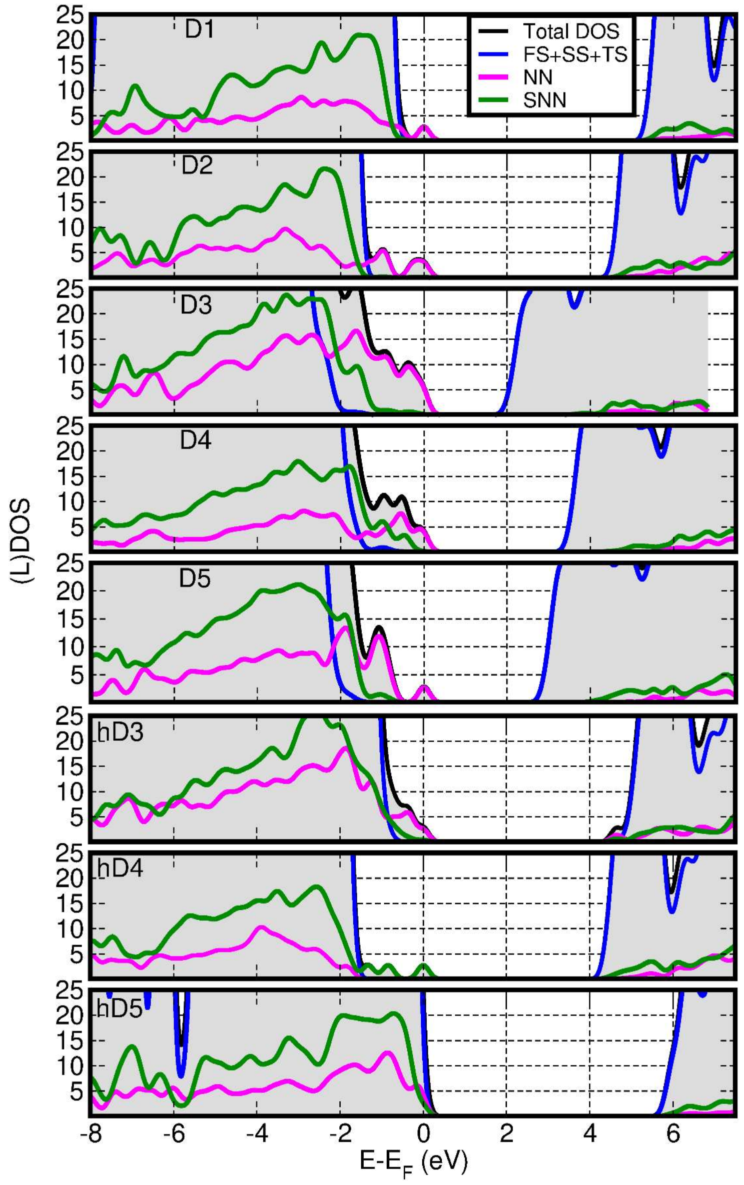

Figure S4. Fermi-energy (E_F) aligned **PBE+U=7 eV** DOS for all the defects simulated (**PBE-optimised geometry**). NN-, SNN- and (FS+SS+TS) resolved local-DOS (LDOS) are also displayed. The energies have been referenced to the highest occupied Kohn-Sham states and the (L)DOS have broadened via 0.1 eV Gaussian smearing. All the computed systems are insulating with no fractional occupancy: spilling of the LDOS traces beyond E_F is due to the applied smearing.

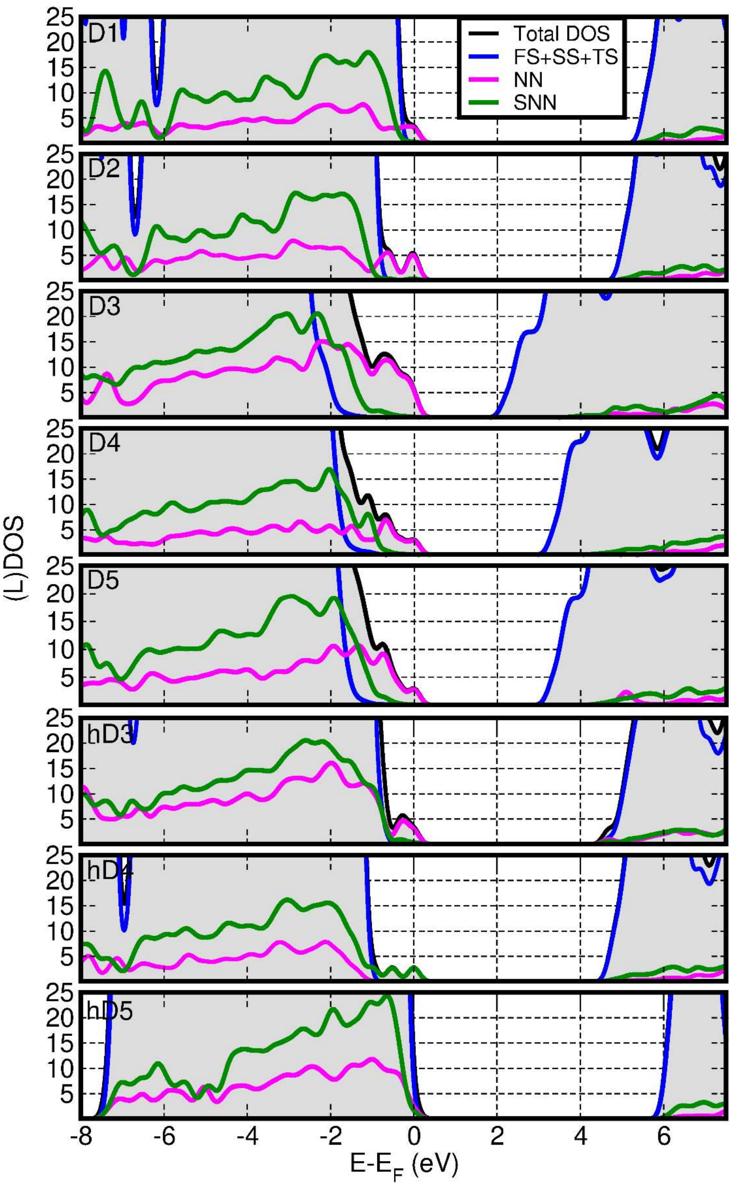

Figure S5. Fermi-energy (E_F) aligned **PBE0-TC-LRC (12.5% HF mixing)** DOS for all the defects simulated (**PBE-optimised geometry**). NN-, SNN- and (FS+SS+TS) resolved local-DOS (LDOS) are also displayed. The energies have been referenced to the highest occupied Kohn-Sham states and the (L)DOS have broadened via 0.1 eV Gaussian smearing. All the computed systems are insulating with no fractional occupancy: spilling of the LDOS traces beyond E_F is due to the applied smearing.

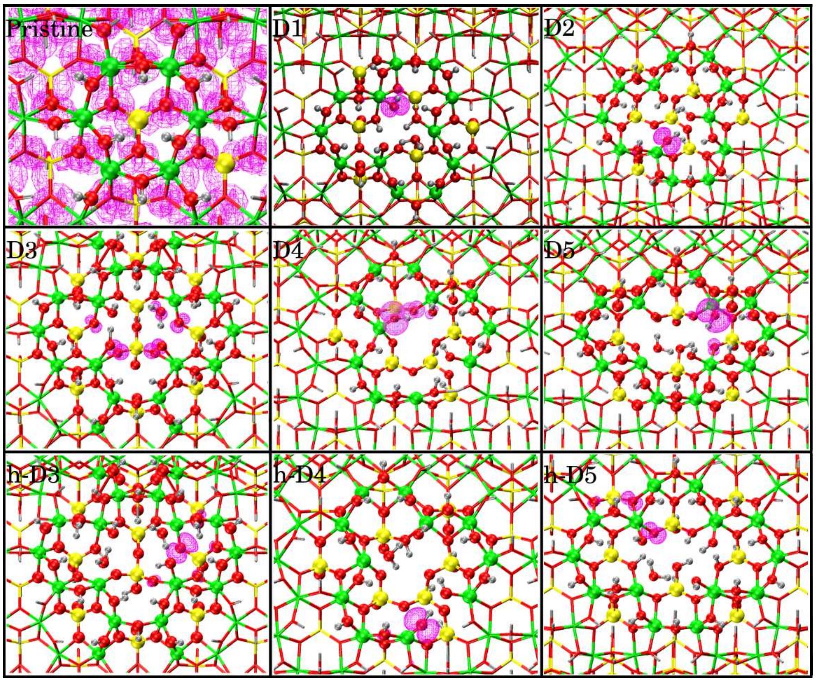

Figure S6. Close up of the **PBE+U=7 eV** density-plot of the highest-energy occupied KS states (magenta). **PBE-optimised geometries**.

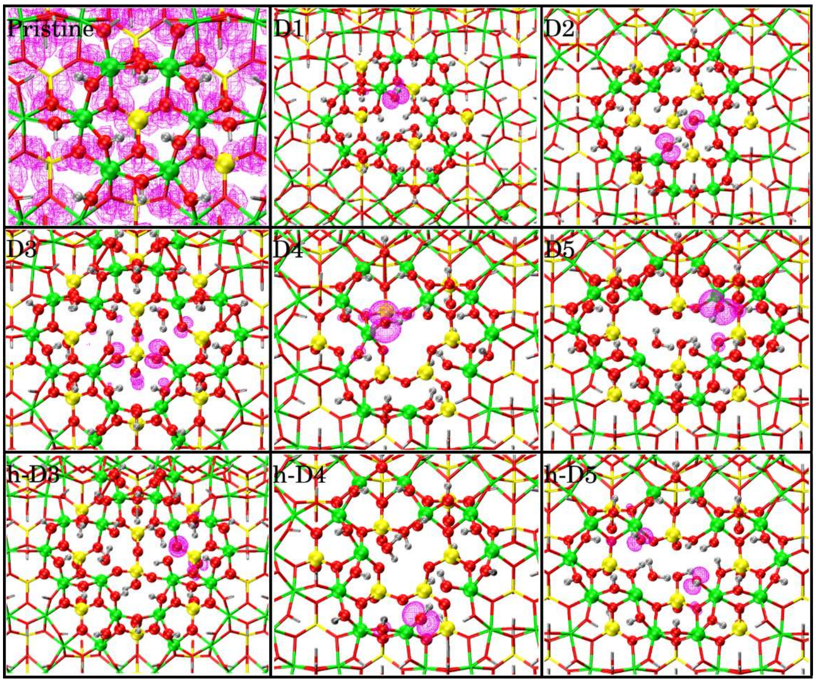

Figure S7. Close up of the **PBE0-TC-LRC (12.5% HF mixing)** density-plot of the highest-energy occupied KS states (magenta). **PBE-optimised geometries**.

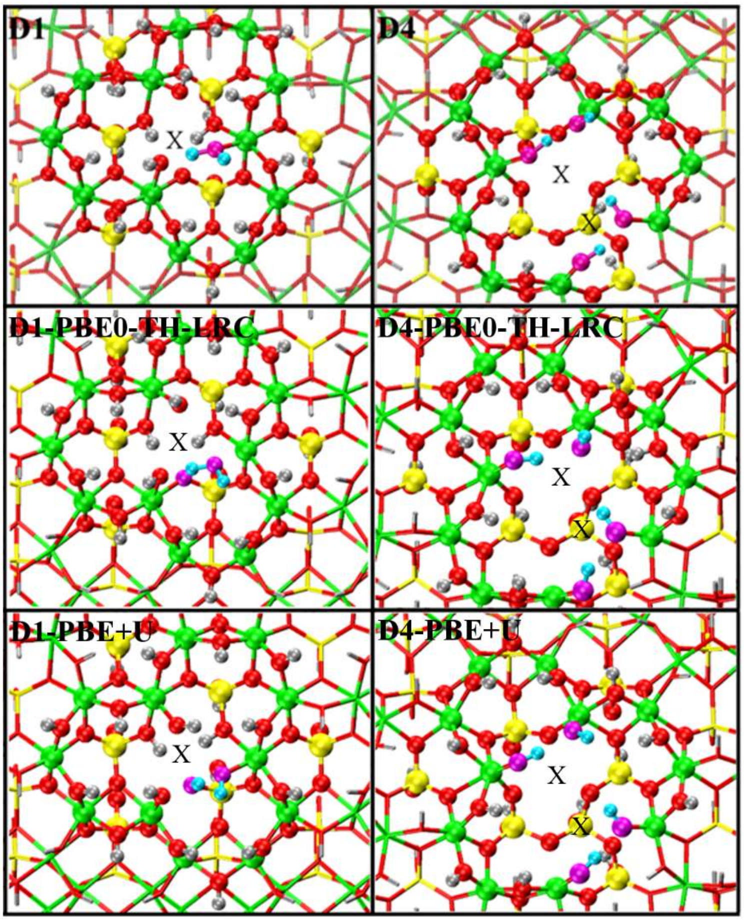

Figure S8. Comparison between the **PBE** (top), **PBE0-TH-LRC** (25% HF mixing, middle) and **PBE+U** (bottom) optimised atomic structures for **D1** and **D4**. Al: green, Si: yellow, O: red, H: silver. To highlight changes from the initial structures in Fig. 1, an alternative atom colouring has been used for the dangling -OH groups closest to the defect sites, condensed H_2O molecules, perturbed hydrogen-bonding patterns. (O: magenta, H: cyan, Si: tangerine). An X marker has been used to indicate the position of the missing (Al or Si) atom in the pristine lattice and of the additional Si atoms.

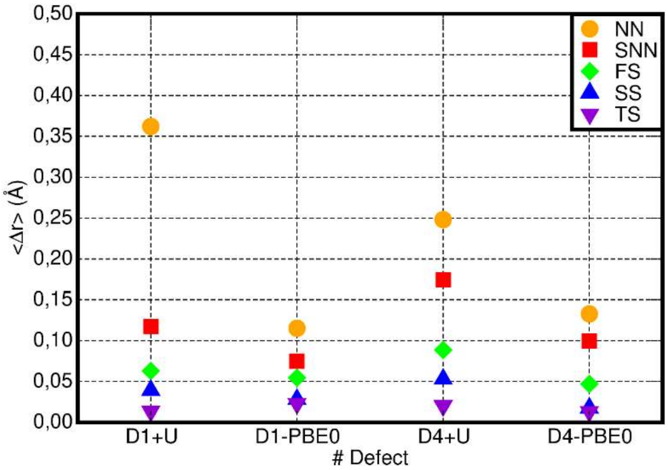

Figure S9. Computed average atomic-displacements **from the PBE** optimised geometry positions (⟨Δr⟩, Å) for the PBE0-TH-LRC (25% HF mixing), and PBE+U optimised atomic structures of D1 and D4. See the manuscript for the definition of the NN, SNN, FS, SS, and TS labelling for the different group of atoms as a function of their distance from centre of the defects.

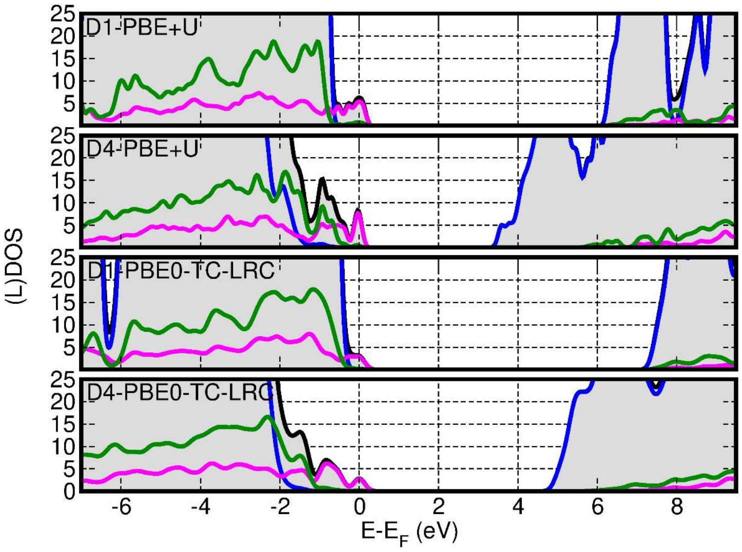

Figure S10. Fermi-energy (E_F) aligned DOS for the **PBE+U** and **PBE0-TH-LRC** optimised atomic structures of D1 and D4. NN-, SNN- and (FS+SS+TS) resolved local-DOS (LDOS) are also displayed. The energies have been referenced to the highest occupied Kohn-Sham states and the (L)DOS have broadened via 0.1 eV Gaussian smearing. All the computed systems are insulating with no fractional occupancy: spilling of the LDOS traces beyond E_F is due to the applied smearing.

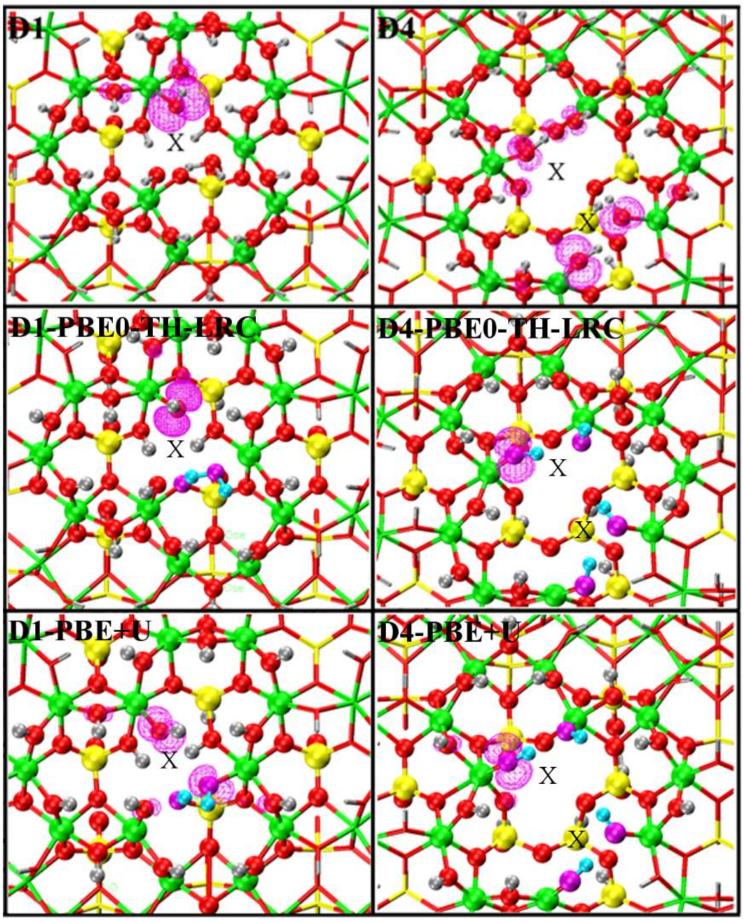

Figure S11. Close up of the density-plot of the highest-energy occupied KS states (magenta) for the **PBE** (top), **PBE0-TH-LRC** (25% HF mixing, middle) and **PBE+U** (bottom) optimised D1 and D4 defects.

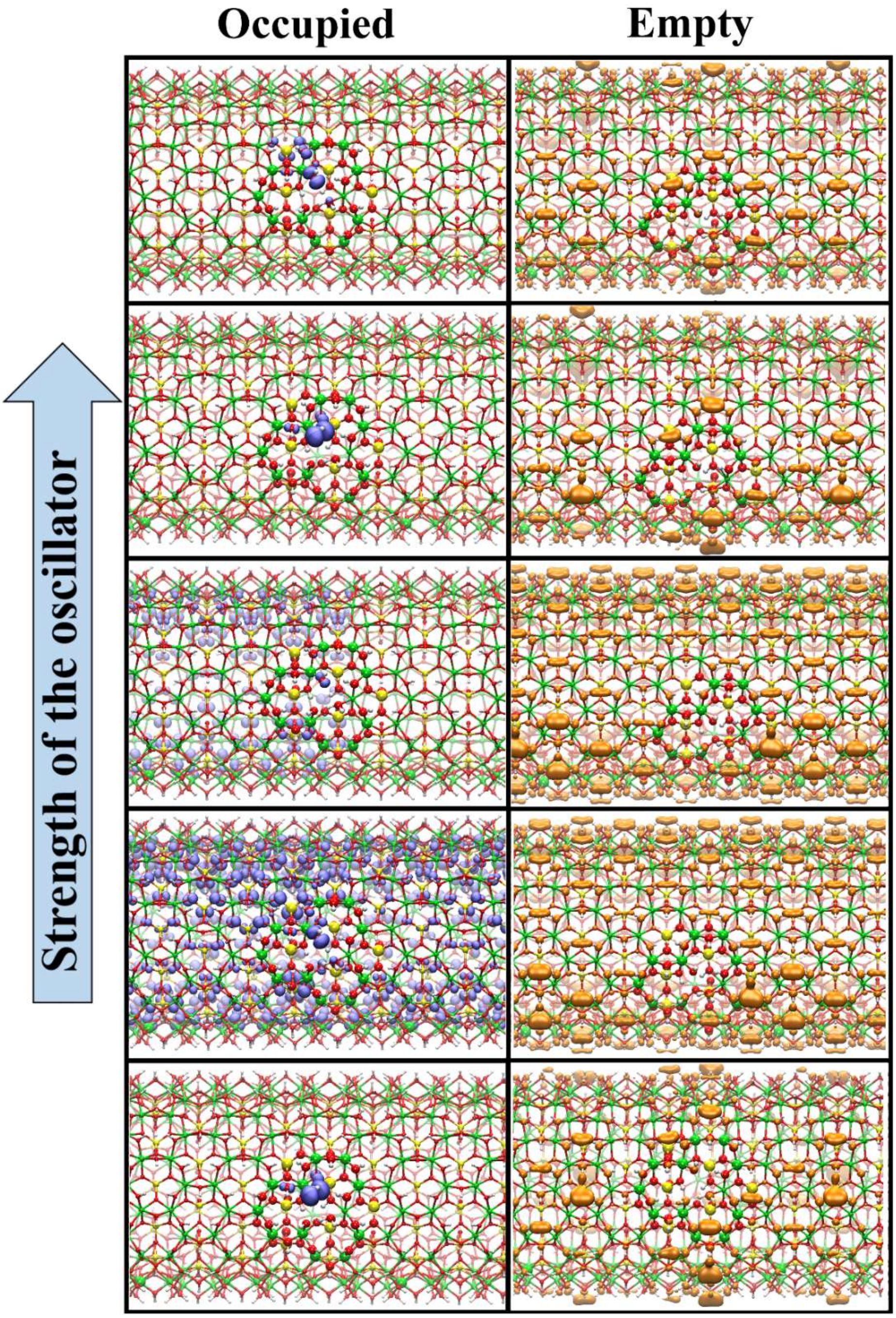

Figure S12. Density plots for the occupied (left) and virtual (right) orbitals involved in the five largest oscillator strength excitations of **D1**. Top row: largest oscillator strength, bottom row: 5th largest oscillator strength.

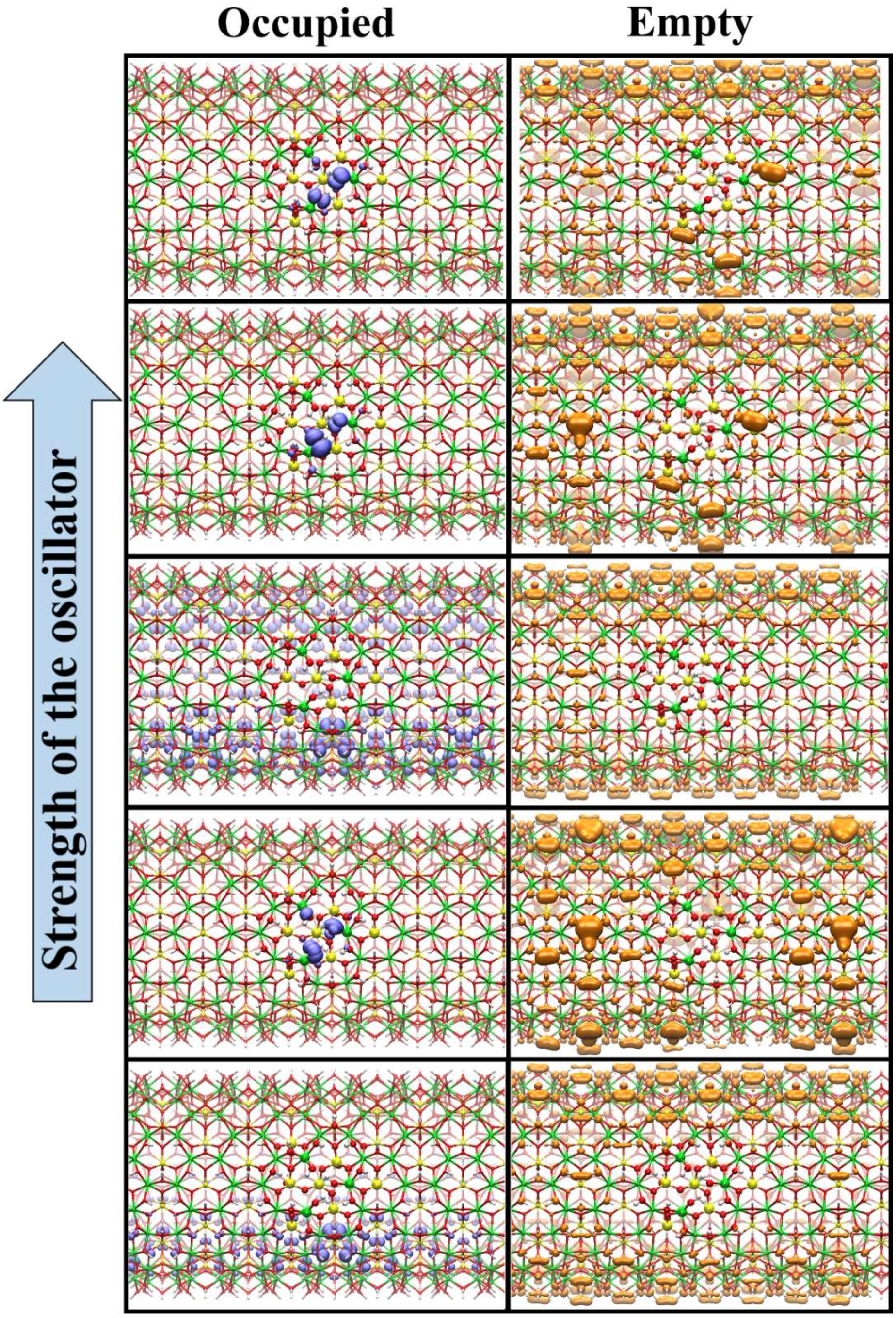

Figure S13. Density plots for the occupied (left) and virtual (right) orbitals involved in the five largest oscillator strength excitations of **D2**. Top row: largest oscillator strength, bottom row: 5th largest oscillator strength.

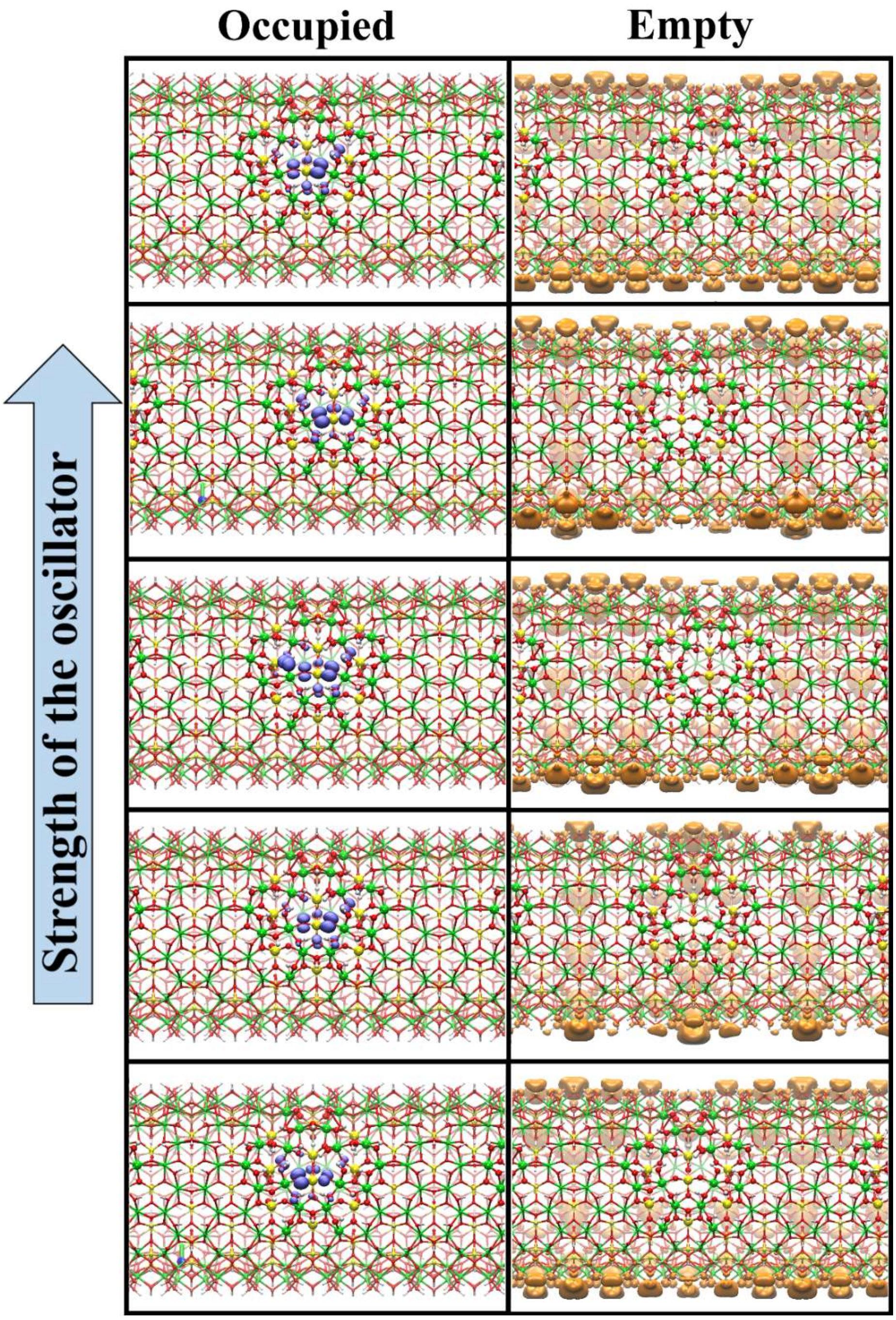

Figure S14. Density plots for the occupied (left) and virtual (right) orbitals involved in the five largest oscillator strength excitations of **D3**. Top row: largest oscillator strength, bottom row: 5th largest oscillator strength.

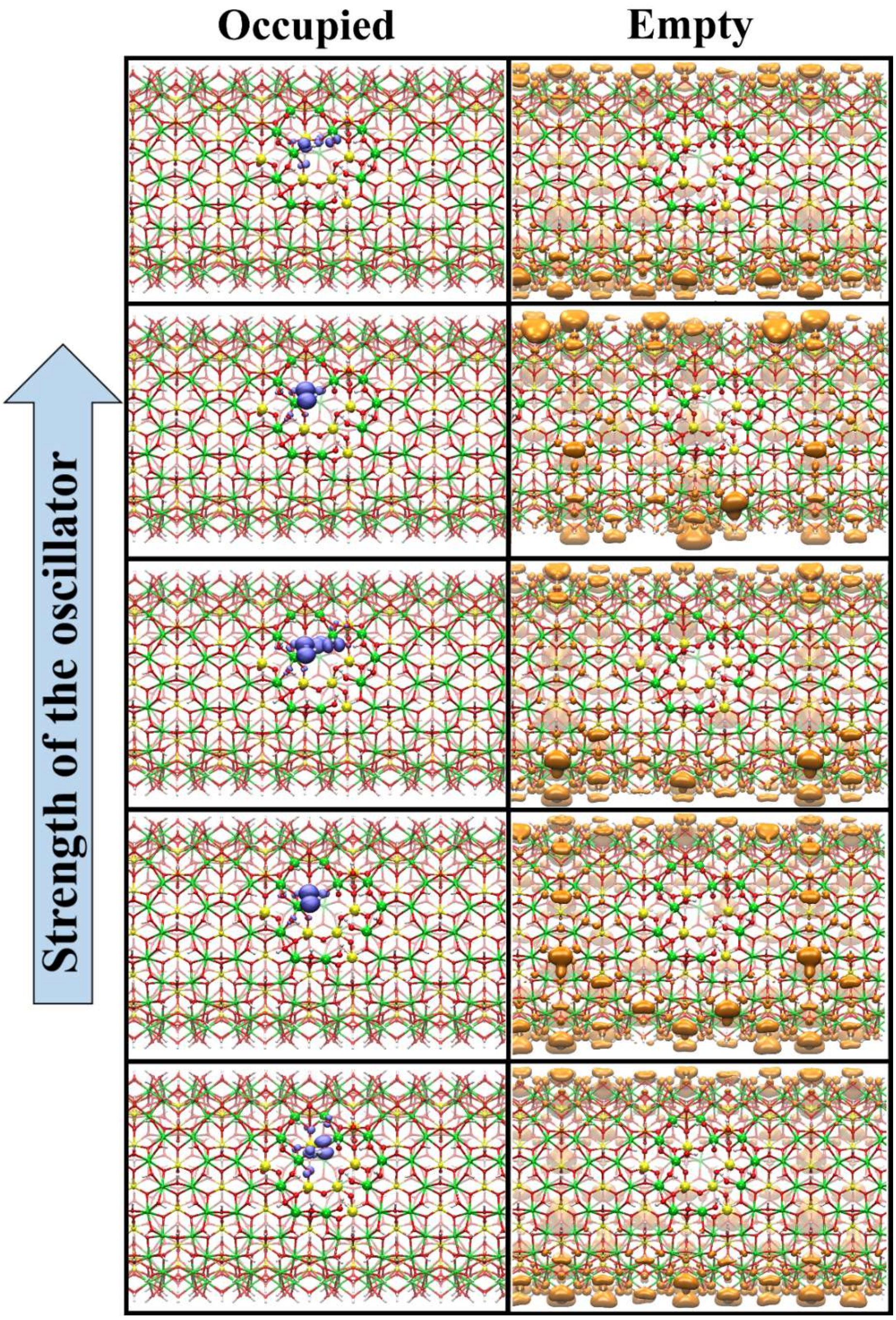

Figure S15. Density plots for the occupied (left) and virtual (right) orbitals involved in the five largest oscillator strength excitations of **D4**. Top row: largest oscillator strength, bottom row: 5th largest oscillator strength.

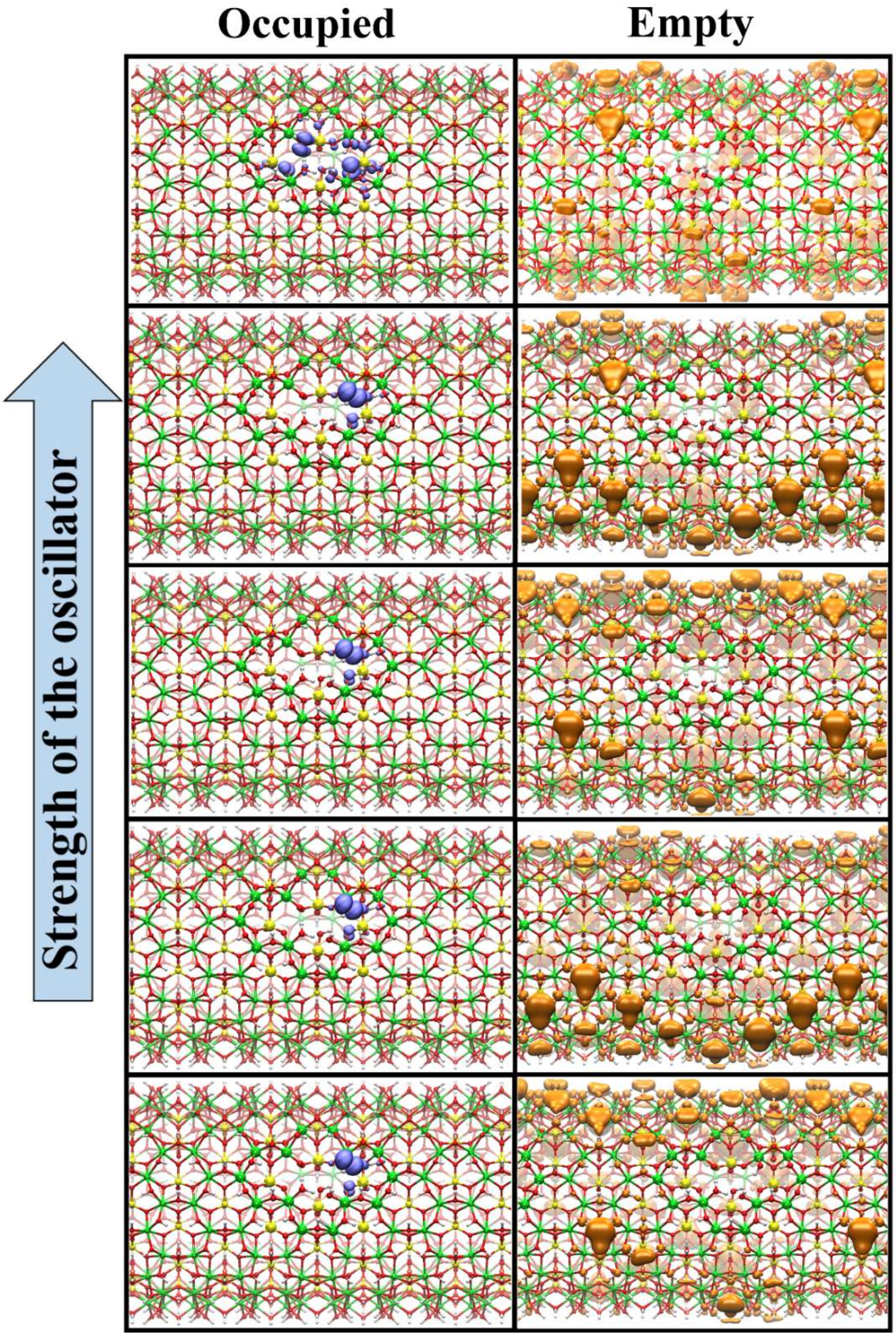

Figure S16 Density plots for the occupied (left) and virtual (right) orbitals involved in the five largest oscillator strength excitations of **D5**. Top row: largest oscillator strength, bottom row: 5th largest oscillator strength.

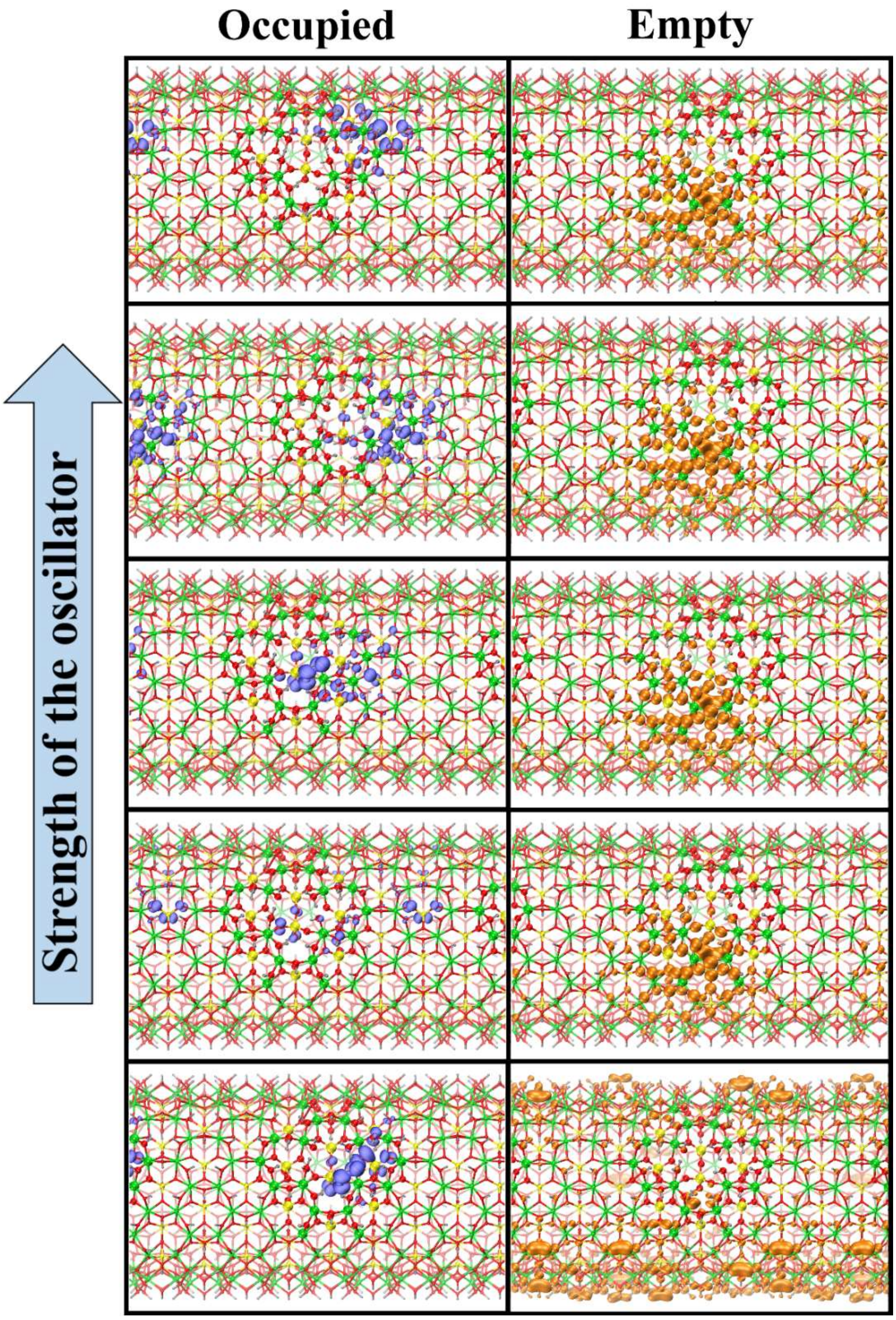

Figure S17. Density plots for the occupied (left) and virtual (right) orbitals involved in the five largest oscillator strength excitations of **h-D3**. Top row: largest oscillator strength, bottom row: 5th largest oscillator strength.

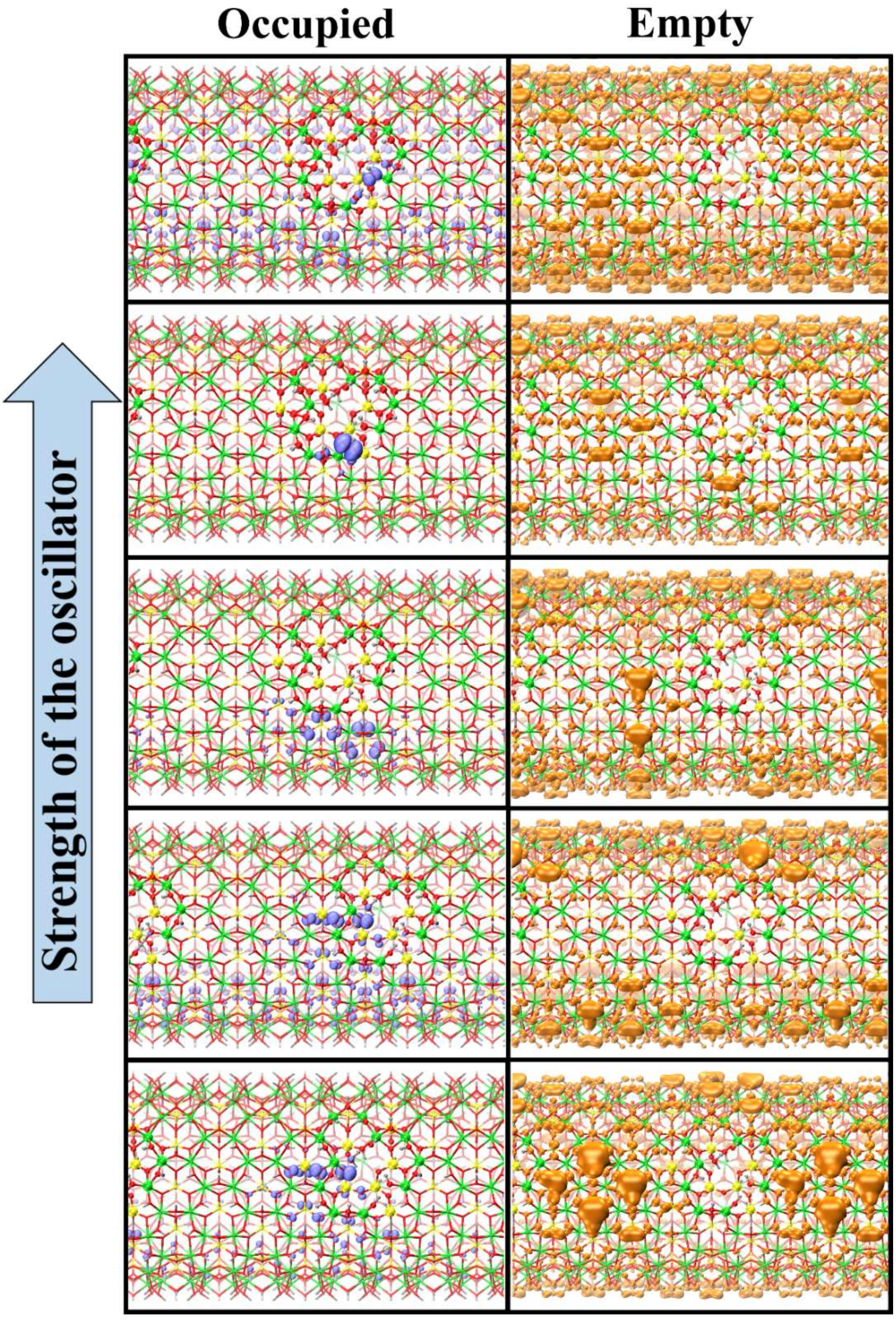

Figure S18. Density plots for the occupied (left) and virtual (right) orbitals involved in the five largest oscillator strength excitations of **h-D4**. Top row: largest oscillator strength, bottom row: 5th largest oscillator strength.

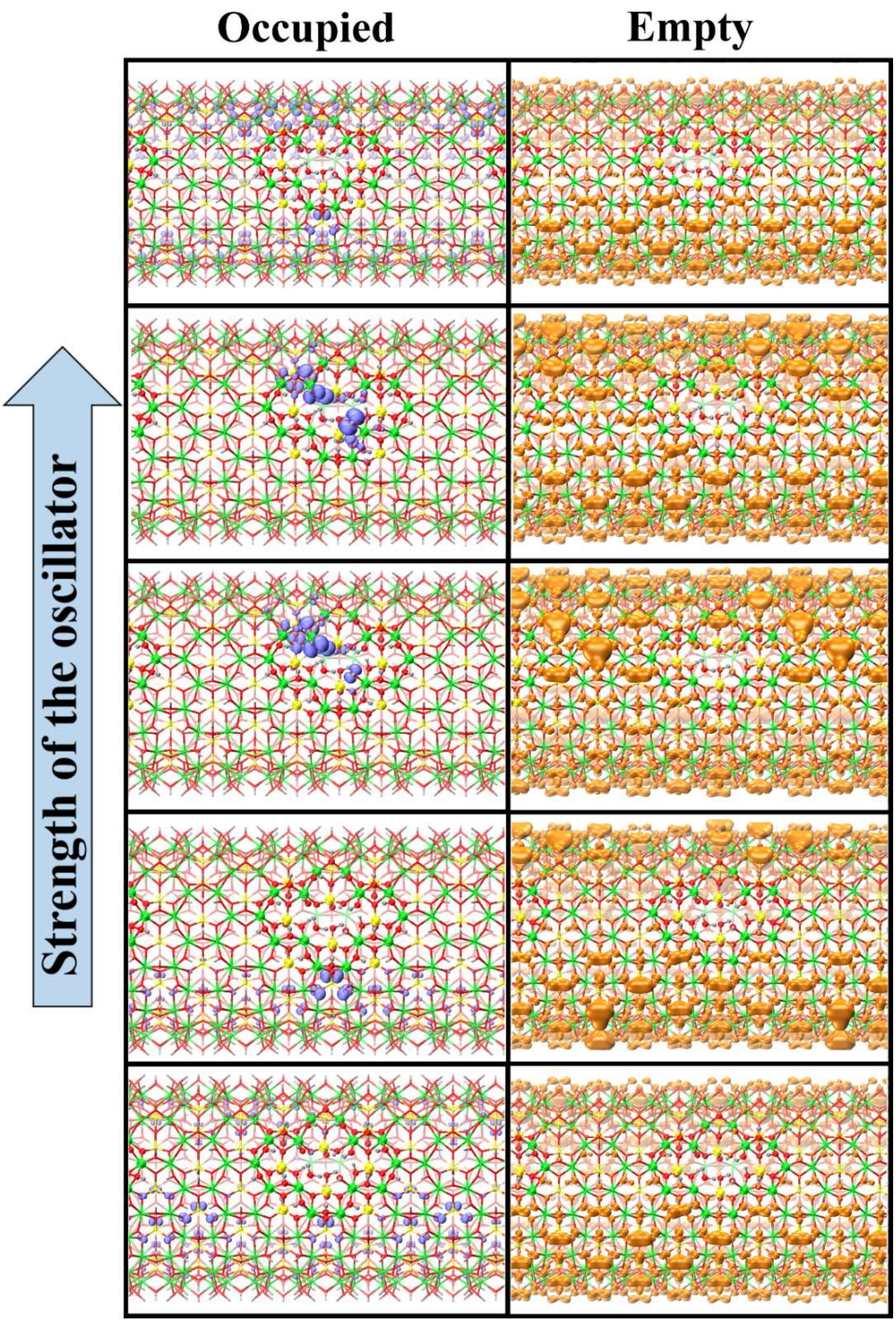

Figure S19. Density plots for the occupied (left) and virtual (right) orbitals involved in the five largest oscillator strength excitations of **h-D5**. Top row: largest oscillator strength, bottom row: 5th largest oscillator strength.

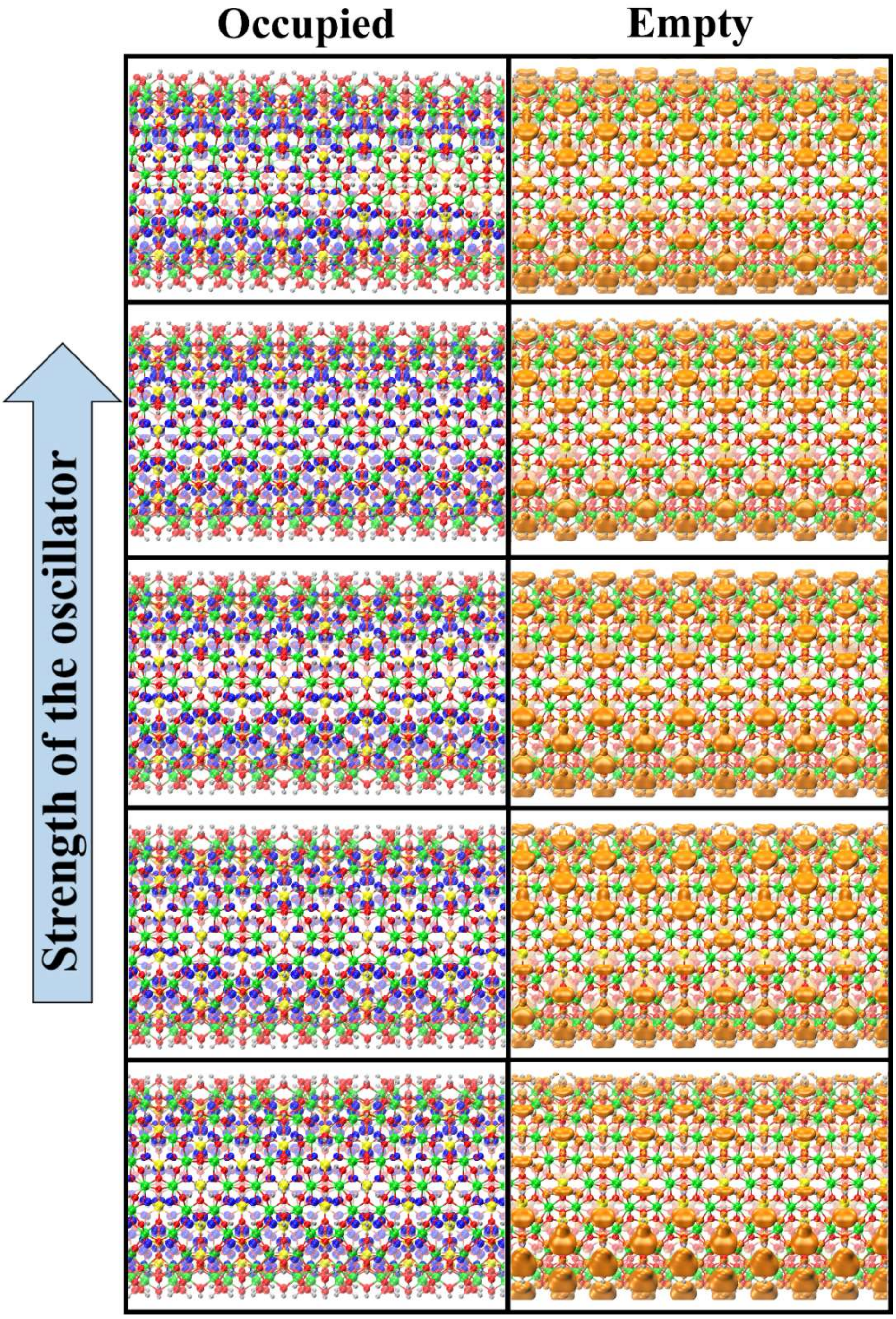

Figure S20. Density plots for the occupied (left) and virtual (right) orbitals involved in the five largest oscillator strength excitations of the pristine **defect-free NT**. Top row: largest oscillator strength, bottom row: 5th largest oscillator strength.

1.2 Supplementary tables.

Bond type	D1	D2	D3	D4	D5
{Si-O} NN	1.65 ± 0.02 Å	1.65 ± 0.01 Å	1.65 ± 0.04 Å	1.65 ± 0.02 Å	1.65 ± 0.03 Å
{Al-O} NN	1.92 ± 0.04 Å	1.87 ± 0.08 Å	1.90 ± 0.05 Å	1.91 ± 0.05 Å	1.90 ± 0.04 Å
{O-H} NN	1.14 ± 0.28 Å	1.22 ± 0.37 Å	1.04 ± 0.16 Å	1.17 ±0.35 Å	1.15 ± 0.35 Å
{Si-O} SNN	1.65 ± 0.01 Å	1.65 ± 0.00 Å	1.65 ± 0.01 Å	1.65 ±0.01 Å	1.65 ± 0.01 Å
{Al-O} SNN	1.92 ± 0.03 Å	1.92 ± 0.03 Å	1.91 ± 0.03 Å	1.91 ± 0.04 Å	1.92 ± 0.03 Å
{O-H} SNN	0.96 ± 0.01 Å	0.96 ± 0.00 Å	0.96 ± 0.01 Å	1.11 ± 0.03 Å	0.96 ± 0.00 Å

Bond type	h-D3	h-D4	h-D5	Pristine
{Si-O} NN	1.64 ± 0.02 Å	1.65 ± 0.03 Å	1.65 ± 0.03 Å	1.65 ± 0.09 Å
{Al-O} NN	1.90 ± 0.02 Å	1.91 ± 0.04 Å	1.91 ± 0.03 Å	1.92 ± 0.16 Å
{O-H} NN	1.09 ± 0.02 Å	1.13 ± 0.32 Å	1.15 ± 0.29 Å	0.96 ± 0.08 Å
{Si-O} SNN	1.65 ± 0.01 Å	1.65 ± 0.01 Å	1.65 ± 0.01 Å	
{Al-O} SNN	1.92 ± 0.03 Å	1.91 ± 0.04 Å	1.92 ± 0.03 Å	
{O-H} SNN	0.96 ± 0.01 Å	1.10 ± 0.32 Å	0.96 ± 0.01 Å	

Table S1: Average bond lengths for the NN and SNN atoms in the defective structures studied and in the pristine NTs.

System	Δ[E_{exc}-KS(BG)] PBE (eV)	Δ[E_{exc}-KS(BG)] PBE0-TC-LRC (eV)
**D1**	0.468	1.074
**D2**	0.442	0.869
**D3**	0.472	0.558
**D4**	0.494	0.476
**D5**	0.508	0.482
**h-D3**	0.420	0.476
**h-D4**	0.434	0.328
**h-D5**	0.425	0.502
**Pristine**	0.465	1.111

Table S2. Computed differences {Δ[E_{exc}-KS(BG)], eV} between the calculated Kohn-Sham BG and LR-TDA-TDDFT lowest energy excitation energy at PBE (left) and PBE0-TC-LRC (right).

D1

PBE										
E	μ	$N_o(1)$	$N_v(1)$	A_1	$N_o(2)$	$N_v(2)$	A_2	$N_o(3)$	$N_v(3)$	A_3
4.9147	0.00470	2016	2039	0.9963	2003	2017	-0.0558			
4.7396	0.00368	2016	2033	-0.9980						
4.8541	0.00322	2014	2019	0.9876	2009	2017	0.1201	2014	2018	0.09565
4.9023	0.00288	2010	2019	-0.9960	2010	2018	-0.0631			
4.3026	0.00285	2016	2019	-0.9959	2016	2018	-0.0852			
HOMO	2016	LUMO	2017							

PBE0-TC-LRC																
E	μ	$N_o(1)$	$N_v(1)$	A_1	$N_o(2)$	$N_v(2)$	A_2	$N_o(3)$	$N_v(3)$	A_3	$N_o(4)$	$N_v(4)$	A_4	$N_o(5)$	$N_v(5)$	A_5
6.8373	0.043560	2014	2019	0.423583	2014	2017	0.3833	2014	2033	-0.2779	2014	2021	0.26666	2014	2027	-0.26047
6.2582	0.031610	2016	2019	-0.47069	2016	2017	-0.3975	2016	2033	0.32606	2016	2027	0.28466	2016	2021	-0.27957
6.9080	0.012980	2012	2017	-0.41814	2012	2019	0.27557	2011	2018	0.27035	2012	2018	0.23141	2011	2017	0.2138
6.9154	0.004970	2010	2017	-0.41828	2009	2018	0.2477	2008	2019	-0.2339	2011	2018	0.20153	2011	2019	0.19948
6.8768	0.003460	2016	2019	-0.69788	2016	2020	0.28505	2016	2027	-0.2819	2016	2018	0.23347	2016	2033	-0.21942

Table S3. Analysis of the five largest oscillator strength (μ) excitations for **D1** at PBE and PBE0-TC-LRC level. The energy of the excitation (E) is reported in eV. For each excitation, the largest amplitude (A_i, i=1,5) transitions between occupied [N_o(i), i=1,5] and virtual [N_v(i), i=1,5] orbitals are also reported.

D2

PBE							
E	μ	$N_o(1)$	$N_v(1)$	A_1	$N_o(2)$	$N_v(2)$	A_2
4.5260	0.00329	2019	2043	0.999465			
4.7151	0.00242	2019	2049	-0.99893			
4.2634	0.00169	2020	2037	-0.99917			
4.3449	0.00166	2019	2037	-0.9975	2018	2022	-0.0627
4.4447	0.00165	2020	2043	-0.99888			
HOMO	2020	LUMO	2021				

PBE0-TC-LRC																
E	μ	$N_o(1)$	$N_v(1)$	A_1	$N_o(2)$	$N_v(2)$	A_2	$N_o(3)$	$N_v(3)$	A_3	$N_o(4)$	$N_v(4)$	A_4	$N_o(5)$	$N_v(5)$	A_5
5.9599	0.011560	2019	2021	0.462015	2019	2023	0.45613	2019	2037	0.29901	2019	2043	0.27515	2019	2025	0.26164
5.8846	0.008960	2020	2023	-0.48554	2020	2021	-0.3953	2020	2037	-0.3022	2020	2025	-0.28641	2020	2043	-0.28088
6.9134	0.007220	2013	2023	-0.29461	2014	2022	0.26318	2012	2021	0.22785	2013	2021	0.22337	2011	2021	0.20784
6.5681	0.005830	2020	2030	0.294076	2017	2021	0.28691	2020	2025	-0.2781	2017	2023	0.26852	2020	2033	0.21918
6.9076	0.004550	2015	2022	0.46465	2015	2023	0.3628	2015	2024	0.27859	2015	2021	0.19042	2014	2024	-0.15851

Table S4. Analysis of the five largest oscillator strength (μ) excitations for **D2** at PBE and PBE0-TC-LRC level. The energy of the excitation (E) is reported in eV. For each excitation, the largest amplitude (A_i, i=1,5) transitions between occupied [N_o(i), i=1,5] and virtual [N_v(i), i=1,5] orbitals are also reported.

D3

PBE				
E	μ	$N_o(1)$	$N_v(1)$	A_1
1.7552	0.00013	2007	2019	1
1.9093	0.00005	2007	2022	0.999992
1.9134	0.00003	2008	2027	0.999992
1.5896	0.00003	2007	2016	-1
1.4132	0.00003	2007	2013	1

HOMO	2008	LUMO	2009

PBE0-TC-LRC							
E	μ	$N_o(1)$	$N_v(1)$	A_1	$N_o(2)$	$N_v(2)$	A_2
4.1639	0.000060	2007	2019	0.999717			
4.3313	0.000030	2008	2027	0.997502			
4.3221	0.000020	2007	2022	-0.97341	2005	2018	-0.2183
4.3743	0.000010	2007	2024	0.999457			
4.0278	0.000010	2008	2019	0.999821			

Table S5. Analysis of the five largest oscillator strength (μ) excitations for **D3** at PBE and PBE0-TC-LRC level. The energy of the excitation (E) is reported in eV. For each excitation, the largest amplitude (A_i, i=1,2) transitions between occupied [$N_o(i)$, i=1,2] and virtual [$N_v(i)$, i=1,2] orbitals are also reported. The transitions not shown have a negligible amplitude (<0.01 in absolute value).

D4

PBE				
E	μ	$N_o(1)$	$N_v(1)$	A_1
3.0394	0.00025	2016	2041	-0.99998
2.8256	0.00018	2016	2033	0.999994
3.2467	0.00014	2016	2055	0.999688
3.3122	0.00012	2016	2058	0.999988
3.2292	0.00012	2014	2027	-0.99997

HOMO	2016	LUMO	2017

PBE0-TC-LRC																
E	μ	$N_o(1)$	$N_v(1)$	A_1	$N_o(2)$	$N_v(2)$	A_2	$N_o(3)$	$N_v(3)$	A_3	$N_o(4)$	$N_v(4)$	A_4	$N_o(5)$	$N_v(5)$	A_5
5.6883	0.002540	2013	2025	0.409101	2015	2035	0.38319	2015	2036	-0.272	2012	2022	-0.25126	2015	2033	-0.24509
5.6186	0.001160	2016	2045	-0.48348	2016	2050	-0.435	2016	2041	-0.3488	2016	2047	-0.28773	2016	2051	-0.21067
5.2366	0.001100	2016	2031	-0.62619	2016	2033	-0.5726	2015	2022	0.34179	2016	2041	-0.16936	2016	2030	0.14705
5.4306	0.000910	2016	2037	0.719169	2016	2041	-0.3956	2016	2040	-0.2598	2016	2033	0.22877	2016	2036	-0.16764
5.6681	0.000760	2014	2025	-0.68901	2014	2027	-0.5452	2014	2026	0.17632	2014	2030	-0.16787	2014	2033	-0.15415

Table S6. Analysis of the five largest oscillator strength (μ) excitations for **D4** at PBE and PBE0-TC-LRC level. The energy of the excitation (E) is reported in eV. For each excitation, the largest amplitude (A_i, i=1,5) transitions between occupied [$N_o(i)$, i=1,5] and virtual [$N_v(i)$, i=1,5] orbitals are also reported.

D5

PBE							
E	μ	$N_o(1)$	$N_v(1)$	A_1	$N_o(2)$	$N_v(2)$	A_2
2.8998	0.00010	2011	2028	-1			
3.0868	0.00005	2011	2038	-0.99991			
3.1029	0.00022	2011	2040	-0.99993			
3.1955	0.00006	2009	2022	-1			
3.2352	0.00008	2011	2046	0.998166	2011	2047	-0.0598
HOMO	2012	LUMO	2013				

PBE0-TC-LRC																
E	μ	$N_o(1)$	$N_v(1)$	A_1	$N_o(2)$	$N_v(2)$	A_2	$N_o(3)$	$N_v(3)$	A_3	$N_o(4)$	$N_v(4)$	A_4	$N_o(5)$	$N_v(5)$	A_5
4.6392	0.01647	2010	2078	-0.59984	2010	2080	0.44989	2009	2078	0.2515	2010	2077	-0.23792	2009	2080	-0.18875
5.4675	0.001080	2011	2033	0.60107	2011	2032	0.34567	2011	2028	-0.2909	2011	2040	-0.29082	2011	2038	0.2658
5.3363	0.000950	2011	2028	0.816744	2011	2026	0.35036	2011	2027	0.25681	2011	2040	-0.14891	2011	2038	0.12461
5.4902	0.000840	2011	2033	0.669907	2011	2040	0.38181	2011	2036	-0.3135	2011	2038	-0.29032	2011	2028	0.18949
5.3211	0.000350	2011	2026	0.819677	2011	2027	0.37419	2011	2028	-0.3577	2011	2025	-0.08572	2011	2040	0.08511

Table S7. Analysis of the five largest oscillator strength (μ) excitations for **D5** at PBE and PBE0-TC-LRC level. The energy of the excitation (E) is reported in eV. For each excitation, the largest amplitude (A_i, i=1,5) transitions between occupied [N_o(i), i=1,5] and virtual [N_v(i), i=1,5] orbitals are also reported.

h-D3

PBE													
E	Oscill.	$N_o(1)$	$N_v(1)$	A_1	$N_o(2)$	$N_v(2)$	A_2	$N_o(3)$	$N_v(3)$	A_3	$N_o(4)$	$N_v(4)$	A_4
4.4350	0.00349	1998	2009	0.999033									
4.5609	0.00169	1991	2009	0.942496	1990	2009	0.32241	1992	2009	-0.0516	2007	2027	-0.05048
3.8047	0.00167	2006	2009	-0.9996									
4.5945	0.00129	2001	2010	-0.71297	2006	2025	-0.698						
4.5670	0.00100	2007	2027	-0.73218	1990	2009	0.62868	1991	2009	-0.2491			
HOMO	2008	LUMO	2009										

PBE0-TC-LRC																
E	μ	$N_o(1)$	$N_v(1)$	A_1	$N_o(2)$	$N_v(2)$	A_2	$N_o(3)$	$N_v(3)$	A_3	$N_o(4)$	$N_v(4)$	A_4	$N_o(5)$	$N_v(5)$	A_5
7.3137	0.01705	1912	2009	-0.28185	1914	2009	-0.2476	1917	2009	-0.2357	2002	2009	0.18536	1981	2009	-0.17897
6.9911	0.012740	1986	2009	0.358611	1999	2009	-0.298	1992	2009	-0.2836	2001	2009	-0.26703	1989	2009	-0.22228
6.9112	0.011810	1992	2009	0.562214	1986	2009	0.21349	2007	2012	0.20802	1989	2009	-0.1989	1990	2009	0.19757
7.3646	0.009200	1933	2009	0.367459	1928	2009	0.2539	1981	2009	-0.2489	1968	2009	-0.22447	1889	2009	-0.18596
7.4247	0.009190	2006	2016	0.323463	2007	2016	-0.27	2006	2015	0.25042	2006	2014	-0.23491	2006	2025	-0.20629

Table S8. Analysis of the five largest oscillator strength (μ) excitations for **h-D3** at PBE and PBE0-TC-LRC level. The energy of the excitation (E) is reported in eV. For each excitation, the largest amplitude (A_i, i=1,5) transitions between occupied [N_o(i), i=1,5] and virtual [N_v(i), i=1,5] orbitals are also reported.

h-D4

PBE							
E	μ	$N_o(1)$	$N_v(1)$	A_1	$N_o(2)$	$N_v(2)$	A_2
4.2195	0.00407	2016	2039	-0.99911			
4.0460	0.00276	2016	2033	-0.99946			
4.7120	0.00148	2014	2021	0.992867	2015	2038	-0.1138
4.5712	0.00135	2014	2019	-0.96587	2015	2033	-0.2581
3.5969	0.00129	2016	2019	-0.99995			
HOMO	2016	LUMO	2017				

PBE0-TC-LRC																	
E	μ	$N_o(1)$	$N_v(1)$	A_1	$N_o(2)$	$N_v(2)$	A_2	$N_o(3)$	$N_v(3)$	A_3	$N_o(4)$	$N_v(4)$	A_4	$N_o(5)$	$N_v(5)$	A_5
7.5184	0.05288	2010	2018	-0.24104	2007	2017	0.23298	2007	2019	-0.23	2005	2017	0.22547	2003	2017	0.19907
6.1804	0.01635	2016	2019	-0.50311	2016	2017	-0.3876	2016	2033	0.38549	2016	2021	-0.31355	2016	2039	-0.30842
7.4935	0.01367	2013	2019	0.500506	2013	2020	0.3622	2013	2018	0.30129	2013	2026	0.2161	2013	2017	0.17237
7.5348	0.01073	2009	2018	0.313143	2011	2017	-0.2579	2011	2019	-0.2263	2005	2018	0.16626	2010	2020	-0.16564
7.5398	0.00913	2011	2017	0.248209	2011	2019	0.24652	2009	2019	0.21361	2009	2018	0.18201	2011	2021	0.16746

Table S9. Analysis of the five largest oscillator strength (μ) excitations for **h-D4** at PBE and PBE0-TC-LRC level. The energy of the excitation (E) is reported in eV. For each excitation, the largest amplitude (A_i, i=1,5) transitions between occupied [$N_o(i)$, i=1,5] and virtual [$N_v(i)$, i=1,5] orbitals are also reported.

h-D5

PBE													
E	μ	$N_o(1)$	$N_v(1)$	A_1	$N_o(2)$	$N_v(2)$	A_2	$N_o(3)$	$N_v(3)$	A_3	$N_o(4)$	$N_v(4)$	A_4
4.8210	0.00426	2010	2015	0.997915	2010	2014	-0.0551						
4.8883	0.00301	2007	2014	-0.99684									
4.7575	0.00230	2010	2013	-0.99928									
4.9727	0.00227	2010	2017	-0.77628	1999	2015	0.61639	1996	2014	0.08756	2012	2020	-0.06749
4.8341	0.00220	2008	2013	-0.99973									
HOMO	2012	LUMO	2013										

PBE0-TC-LRC																
E	μ	$N_o(1)$	$N_v(1)$	A_1	$N_o(2)$	$N_v(2)$	A_2	$N_o(3)$	$N_v(3)$	A_3	$N_o(4)$	$N_v(4)$	A_4	$N_o(5)$	$N_v(5)$	A_5
7.5163	0.05434	2007	2013	-0.37586	2007	2014	-0.2745	2009	2013	0.26141	2009	2014	0.25104	2006	2014	0.24031
7.3089	0.05047	2012	2015	-0.29581	2011	2013	0.25704	2011	2015	0.25283	2012	2013	-0.21369	2011	2014	-0.2045
7.3978	0.02584	2010	2015	0.408159	2010	2013	0.30488	2010	2029	0.26359	2012	2015	-0.25918	2010	2017	0.24634
7.4978	0.01450	2008	2014	-0.49595	2008	2013	0.47713	2008	2016	0.22823	2008	2021	-0.17568	2008	2019	0.13829
7.5514	0.00455	2001	2013	0.341554	2001	2014	-0.2709	2002	2013	0.26527	1998	2014	0.20159	2004	2014	0.17787

Table S10. Analysis of the five largest oscillator strength (μ) excitations for **h-D5** at PBE and PBE0-TC-LRC level. The energy of the excitation (E) is reported in eV For each excitation, the largest amplitude (A_i, i=1,5) transitions between occupied [$N_o(i)$, i=1,5] and virtual [$N_v(i)$, i=1,5] orbitals are also reported.

Pristine	PBE													
	E	μ	$N_o(1)$	$N_v(1)$	A_1	$N_o(2)$	$N_v(2)$	A_2	$N_o(3)$	$N_v(3)$	A_3	$N_o(4)$	$N_v(4)$	A_4
	4.8942	0.00816	2015	2019	0.583953	2014	2018	-0.5693	2014	2019	0.4227	2015	2018	0.39481
	4.8347	0.00535	2016	2017	-0.99983									
	4.8927	0.00208	2015	2018	0.593008	2014	2019	0.5606	2014	2018	0.41529	2015	2019	-0.40192
	4.9275	0.00003	2010	2019	0.773379	2011	2018	0.61249	2010	2018	-0.146	2011	2019	-0.06872

HOMO	2016	LUMO	2017

Pristine	PBE0-TC-LRC																
	E	μ	$N_o(1)$	$N_v(1)$	A_1	$N_o(2)$	$N_v(2)$	A_2	$N_o(3)$	$N_v(3)$	A_3	$N_o(4)$	$N_v(4)$	A_4	$N_o(5)$	$N_v(5)$	A_5
	6.9107	0.01424	2016	2017	0.466441	2015	2018	-0.3493	2014	2019	0.34733	2013	2021	-0.25601	2012	2020	0.24679
	6.9592	0.00015	2009	2017	-0.43799	2011	2019	-0.2848	2010	2018	-0.2848	2012	2020	-0.20618	2013	2021	-0.20226
	7.0627	0.00003	1958	2017	0.350503	1964	2018	-0.2018	1963	2019	0.2001	1940	2020	0.19183	1969	2021	-0.17413
	6.9139	0.00003	2014	2017	-0.45946	2016	2019	-0.3787	2013	2018	0.27316	2012	2019	-0.26077	2015	2021	0.20053
	6.9139	0.00003	2015	2017	-0.45958	2016	2018	0.37904	2013	2019	-0.2724	2012	2018	-0.26108	2014	2021	0.20005

Table S11. Analysis of the five largest oscillator strength (μ) excitations for the pristine **defect-free NT** at PBE and PBE0-TC-LRC level. The energy of the excitation (E) is reported in eV. For each excitation, the largest amplitude (A_i, i=1,5) transitions between occupied [N_o(i), i=1,5] and virtual [N_v(i), i=1,5] orbitals are also reported.
